# Supplementary material for: Germline AGO2 mutations impair RNA interference and human neurological development
Source: Nat Commun. 2020 Nov 16;11:5797. doi: 10.1038/s41467-020-19572-5 (PMC7670403; doi:10.1038/s41467-020-19572-5)
Supplement: Supplementary file 1 — Supplementary Information [file 41467_2020_19572_MOESM1_ESM.pdf]

Supplementary Information

Germline *AGO2* mutations impair RNA interference and human neurological development

D. Lessel et al.

Supplementary Note 1

Case 1

This 13 months old male is the third child of non-consanguineous healthy Russian parents. The family history was unremarkable. His older siblings are unaffected. He was born at 38 weeks gestation with a birth weight of 2745 gram (-1.3 SD), birth length was 47 cm (-1.8 SD) and his head circumference was 35 cm (+0.1 SD). APGAR scores were 6 and 7 after 1 and 5 minutes respectively as there was a perinatal complication of cord around the baby's neck. He was diagnosed with congenital lobar emphysema for which a thoracotomy with resection of the left upper lobe was performed. At the last clinical examination at 13 months he had motor delay and was unable to sit. He also had oral sensitivity with refusal to bottle and reduced babbling. His height was 73.5 cm (-1.4 SD), weight was 7.1 kg (-1.8 SD) and head circumference of 46.5 cm (-0.7 SD). He had prominent cheeks and retrognathia. He also had muscular hypotonia. Computed Tomography of the brain at 13 months old was normal. Metabolic screening and array CGH were normal. Trio-whole exome sequencing revealed a *de novo AGO2 in-frame* deletion: Chr8(GRCh37):g.141570582_141570584del; NM_001164623.2:c.544_546del, p.(Phe182del). The *de novo* occurrence was confirmed by Sanger sequencing in the patient and both parents.

Case 2

This 4.5 year old male was born as the first child of healthy non-consanguineous parents. The family history was unremarkable. He has a younger unaffected sister. He was born after 41+1 weeks of gestation by a vacuum-assisted vaginal delivery. His birth weight was 2980 gram (-1.7 SD), birth length was 49cm (-1.8 SD) and his head circumference was 38 cm (+1.6 SD). APGAR scores were 8, 9 and 10. He required cardiopulmonary resuscitation in the first hour of life and respiratory support with BIBAP for 4 hours. Cardiac investigation revealed patent ductus arteriosus and patent foramen ovale. Brain MRI at the age of 3 weeks and 11 months revealed hypoplasia of corpus callosum and absent septum pellucidum but no other abnormalities and age-appropriate myelinisation. The early postnatal period was characterized by feeding difficulties, recurrent central apnea and severe truncal muscular hypotonia. Cryptorchidism of the left testicle was repaired by surgery at the age of one year. The boy developed a skull deformity defined as a combination between brachy- and plagiocephaly. Motor development is severely delayed, he learned to crawl at the age of 3 years, and can make 2-3 steps alone using orthoses at the age of 4.5 years. He doesn´t speak and has poor receptive language. He developed four febrile seizures, last at the age of 4.5 years, but epileptic discharges are absent in several EEG so far. Eye examination by an ophthalmologist revealed a strabism and hyperopia. Last physical examination at the age of 4 years and 4 months revealed a friendly boy with severe global developmental delay with truncal muscular hypotonia and mild lumbal scoliosis. His weight was 15.7 kg (-0.9SD), height was 98.5 cm (-1.9SD) and head circumference was 52 cm (+0.5SD). He had facial dysmorphisms including epicanthic folds, upslanting palpebral fissures, helix hypoplasia, thin upper lip, prominent forehead and open mouth appearance with extensive salivation. Trio-whole exome sequencing revealed a *de novo AGO2* mutation: Chr8(GRCh37):g.141570553T>C; NM_012054.3:c.575A>G, p.(Leu192Pro). The *de novo* occurrence was confirmed by Sanger sequencing in the patient and both parents.

Case 3

This 17.5 year old female is the only full biological child to healthy non-consanguineous parents. The family history is remarkable for a maternal half-brother who has a history of sensory issues and hypermobility. She was born at 42 weeks following a normal spontaneous vaginal delivery. Her birth weight was 3685 gram (+0.1SD), birth length was 52.07 cm (-0.2 SD), and head circumference was 37.5 cm (+1.6 SD). She had jaundice requiring phototherapy. At that time, she was also noted to be lethargic

and her blood glucose level was low necessitating gavage feeding. She was subsequently diagnosed with central apnea, and gastroesophageal reflux. Developmental delays were noted early on in life. She sat supported at 6 months, rolled over at 8 months and took her first steps at 3 years of life. However, her physical motor skills were significantly delayed and she is currently wheelchair-bound. Verbal and language development were severely delayed as she is nonverbal and uses gestures and variably assistive devices for communication. Her medical history has been complex with multiple medical issues including dysmorphic facial features (thin upper lip, deep-set eyes, broad nasal bridge, upslanted palpebral fissures, frontal bossing, open mouth appearance with excessive salivation, somewhat coarse facial features with prominent widow's peak, bitemporal balding, high-arched palate), mild thoracic asymmetry, nevus flammeus on the lower neck and lower back, single palmar creases, bilateral 2-3 toe syndactyly, bilaterally short fingers and toes. She also had plagiocephaly in infancy, in her teens the head shape was rather brachycephalic. She developed seizures consistent with Lennox-Gastaut syndrome. Her current height at age 17 ½ years is 152.4 cm (-2.4 SD) and her weight is 48.9 kg (-1.4 SD). She has not yet completed puberty. She has very limited receptive language. She can respond appropriately implying some receptive language but it is not reliable. She will self-harm by scratching herself or pulling her own hair. She is aggressive with pinching, scratching, biting others, particularly when agitated but sometimes this behavior is to get a reaction out of others. She has some anxiety as she is sensitive and fearful of falling down or anxious when in an unfamiliar location. Brain MRI revealed mildly prominent ventricles with dysplastic corpus callosum, but no other abnormalities. She also developed volvulus status post-surgical repair as well as G-tube placement. Cardiac evaluations were notable for short QT interval, with a normal echocardiogram. Previous genetic evaluations include karyotype (600-650 band resolution) that showed a 46,XX karyotype, chromosomal microarray which was normal, buccal FISH for 12p tetrasomy which showed 8% mosaicism with 300 cells analyzed. Skin fibroblasts were subsequently tested by FISH for tetrasomy of 12p with 100 cells analyzed and were found to be normal. She also underwent several metabolic tests which were all normal. Finally, she underwent trio exome sequencing that revealed a *de novo AGO2* mutation*:* Chr8(GRCh37):g.141570553T>C; NM_012054.3:c.575A>G, p.(Leu192Pro).

Case 4

This female patient is the only child of possible consanguineous parents of Turkish ancestry. There were no developmental problems reported in the family. She was born after 39 weeks of gestation via primary Caesarean section because of breech position. Her birth weight was 1885 gram (-3.5 SD) and her head circumference was 32.8 cm (-2 SD). APGAR scores were 8 and 10 after 1 and 5 minutes, respectively. As neonate, she had severe feeding difficulties requiring tube feeding. Cardiac investigation revealed mild pulmonary valve stenosis and blood analysis showed microcytic anemia due to iron-deficiency. Both motor and language development were delayed. At the age of 2 years, she walked her first steps but was not able to speak. Eye examination by an ophthalmologist revealed an optic pit of the left eye with possible low vision and cerebral visual impairment. Physical examination at the age of 1 year showed normal head circumference of 44.6 cm (-1.3 SD) and at the age of 2.5 year low height of 83 cm (-2.5 SD) and weight of 9.91 kg (-1.8 SD) were measured. She had facial dysmorphisms including frontal bossing, epicanthal folds, broad nasal bridge and thin upper lip. There was mild axial hypotonia present. There were no noticeable abnormalities of the extremities. Brain MRI at the age of 1.5 years showed focal white matter abnormalities. Previous investigations, consisting of MLL2 sequence analysis and H19 methylation analysis, were normal. SNP array showed a paternally inherited duplication 6p21.31(33,903,076-34,062,760)x3 and 9% homozygosity (in line with the possible parental consanguinity). Trio-whole exome sequencing revealed a *de novo AGO2* mutation: Chr8(GRCh37):g.141570527C>A; NM_012054.3:c.601G>T, p.(Gly201Cys).

Case 5

This girl is the only child of unrelated healthy parents (mother 26 years, father 29 years). There was no family history of congenital malformations or autistic spectrum disorder (ASD) or psychiatric disease. She was born via caesarian section for intrauterine growth retardation and fetal distress at 37+5 weeks of gestation, being small for gestational age with 2230 gram (-2,320 SDS) and 45 cm (-2,373 SDS) body length, while head circumference appeared relatively macrocephalic with 33 cm (19. percentile). For poor feeding and muscular hypotonia she was monitored on intensive care unit and diagnosed with mild aortic stenosis in association to biscupid aortic valve. Dysmorphic signs were noted, including epicanthal folds, blue sclerae, high arched palate, bright and brittle hair, and relative macrocephaly. During infancy cryptogenic heart block II-III° occurred with a mean heart frequency of 45/min, which later in childhood led to pacemaker implantation. She was also seen for abnormal breathing pattern with hypopnea and severe food aversion, gastroesophageal reflux and constipation and temporary cow's milk protein free diet was introduced for cow's milk protein allergy. From 3 years on she experienced weekly to monthly focal clonic seizures with secondary generalization, and sensitivity to sunlight necessitated wearing of sunglasses. Teeth discoloration likely occurred because of gastroesophageal reflux, and dental plaque formation occurred due to inability to sustain lip closure with continuing hypersalivation. She suffers from malnutrition and insufficient weight gain, mostly due to inappetence as she refuses meal after taking few spoons. Her current height at age 11 years and 9 months is 118 cm (-4.5 SD), her weight is 19.6 kg (-4.5 SD) and OFC is 51.3 cm (-1.7 SD). Up to the age of 4 years the patient was tested for inherited disorders of metabolism (including analysis of plasma amino acids, urine organic acids, copper, coeruloplasmine, glycosylation defects, very long chain fatty acids) and for multiple genetic syndromes including microdeletion 1p36, 22q11.2, Silver-Russel syndrome (UPD7, 11p15 Epimutation), Williams-Beuren syndrome, Smith-Lemli-Opitz syndrome, Prader-Willi syndrome, Noonan syndrome and related syndromes (PTPN11, KRAS, BRAF, MEK1/2) and myotonic dystrophy. Oligo-SNP-Array (Affymetrix SNP-Chip 6.0) did not identify unknown copy number variations (CNV) and panel evaluation for epilepsy related genes was non-conclusive. Trio whole exome sequencing eventually identified a *de novo AGO2* mutation: Chr8(GRCh37):g.141570526C>A; NM_012054.3:c.602G>T, p.(Gly201Val).

Case 6

This 1.5-year old girl is the fifth child of nonconsanguineous Dutch parents. After a pregnancy of 38 weeks complicated by intrauterine growth retardation, she had a birthweight of 2300 gram (-2SD) and a birth length of 40 cm (-2,5 SD). There were neonatal feeding problems for which nasogastric tube feeding was necessary. Feeding has been troublesome ever since, because of insufficient muscular tone, gastroesophageal reflux and aversion of stimulation around the mouth. From the age of 6 months she had several upper and lower airway infections for which hospital admission was necessary and she is now solely fed by gastric tube. Both motor and speech development are delayed. At 1.5 years, she has incomplete head balance and is not able to roll over. She is not able to speak, but can make eye-contact incidentally. She has a stable strabismus of the left eye since the age of 5 months. Hearing is normal. From the age of 6 months, she seems to have multiple absences during the day. No anticonvulsive medication has been started. She sleeps a lot, but there are no signs of narcolepsy.
Physical examination at the age of 5.5 months showed a short stature of 60 cm (-2,42 SD), a low weight of 4485 gram (-2,4 SD) and a head circumference of 41 cm (-1,35 SD). Dysmorfic features were: scaphocephaly with open skull sutures, large fontanel, frontal bossing, epicanthal folds, upslant, thin upper lip, short toes, proximal implant dig III-IV left foot. There was evident axial hypotonia with complex head lag. Reflexes were normal. At the age of 1 year and 6 months, she has a height of approximately 74 cm (-2.4 SD) and weight of 9120 grams (-1.4 SD). Cardiac evaluation was normal. The MRI showed delayed myelinisation of the genu of the corpus callosum. Opthalmologic evaluation shortly after birth was normal. Metabolic screening and CK in blood were normal. Genetic testing for

CNVs (SNP-array), Prader Willi syndrome, spinal muscular atrophy (SMA) was normal. Trio whole exome sequencing identified a heterozygous *de novo* *AGO2* mutation: Chr8(GRCh37):g.141570526C>A; NM_012054.3:c.602G>T, p.(Gly201Val).

Case 7

This male patient is the second child of non-consanguineous parents of Dutch ancestry. There were no developmental problems reported in the family. He was born after 42+1 weeks of gestation. Labour was induced because of serotinity. APGAR scores were 9 and 10 after 1 and 5 minutes respectively. At the age of six weeks his weight was 5800 gram (+2.0 SD), length was 53 cm (-0.2 SD) and his head circumference was 42 cm (+2.5 SD). As a baby he was quiet and slept a lot. At the age of 5 months, a delayed development became obvious. Brain MRI at the age of 16 months showed a benign external hydrocephalus and a small pituitary gland, but laboratory screening of the pituitary hormones was normal. He started walking at the age of 25 months and at the age of 3 years and 10 months he spoke two-word sentences. At that time his developmental age was 18 months. He was diagnosed with PDD-NOS and eye examination revealed strabismus of the right eye and an end-point nystagmus. Mild symptoms of hyperphagia were present. Physical examination at the age of 30 months showed a normal height of 93.8 cm (0 SD), normal weight of 16 kg (+1.0 SD) and normal head circumference of 53 cm (+1.7 SD). He had a plagiocephaly and deep-set eyes. At 6 years and 10 months he had a height of 123,1cm (-0,4SD) and weight 26,8kg (+1,07SD). He had a slow progression in development. He spoke only short sentences, with smoother and more varied motor skills. Dribbling walk, mostly at the forefoot / toes. He developed autistic features. Metabolic screening, array CGH (180K) and DNA analysis of CGG repeat length of the *FMR1* gene showed normal results. Using trio-whole exome sequencing a *de novo* *AGO2* mutation was identified: Chr8(GRCh37):g.141570519A>T; NM_012154.3:c.609T>A, p.(His203Gln).

Case 8

This 18 year old male was born as the first child of healthy non-consanguineous parents. The family history was unremarkable. He has younger unaffected sister and brother. He was born after 42 weeks of gestation. His birth weight was 4160 gram (+0.78 SD), birth length was 55 cm (+0.67 SD) and his head circumference was 35 cm (-0.79 SD). APGAR scores were normal. The early postnatal period was characterized by feeding difficulties, without muscular hypotonia. Motor development was within the margin, he was able to walk without support at 18 months. Language development was mildly delayed. Last physical examination at the age 16 years revealed a friendly boy with mild global developmental delay. He was diagnosed with delayed fine and gross motor skills by the age of four years of age. He was clumsy and his gait was not fluent till the age of approximately twelve. Although he was never officially diagnosed with autism, parents recognize some symptoms. For example, it is difficult for him to change his normal day routine. He is able to speak in full sentences, although he makes a lot of grammatical errors. Both this boy and his mother (without developmental problems) showed pronounced leg edema. At the age of 16 years his weight was 60.3 kg (+0.1SD), height was 178 cm (+0.2SD) and head circumference was 55 cm (-0.3SD). He had some facial dysmorphisms including elongated face, full upper eyelids, broad mouth and long philtrum. Trio-whole exome sequencing revealed a heterozygous *AGO2* mutation: Chr8(GRCh37):g.141566342G>A; NM_012154.4:c.1070C>T, p.(Thr357Met). The unaffected mother is a mosaic carrier of this mutation, which is present in approximately 15% of her cells.

Cases 9 and 10

This male patient (case 9) is the first child of nonconsanguineous Caucasian parents. He was born at 38 weeks and four days after an on eventful pregnancy. There were no exposures to teratogens or significant maternal health concerns (no gestational diabetes or hypertension). Birth weight was 4422 g (+2.6 SD). Shortly after birth he experienced pulmonary congestion and required CPAP and hewas noted to have low tone. He was discharged from the hospital after seven days.

Development milestones were delayed. He showed a head lag, delayed independent sitting, and he walked at 2 1/2 years. He was described as “clumsy”. His first words were at four years of age. He also showed in-coordination with feeds and fine motor skills.

He experienced several febrile seizures. He was diagnosed with obstructive sleep apnea at age 5 years. He required a CPAP at night until an adenoid and tonsillectomy resolved the apneic episodes. He has a long-standing history of constipation and requires a laxative. An MRI performed at 4 months of age given concerns of developmental delay and macrocephaly. The MRI showed a thin corpus callosum and prominent extra-axial spaces. A skeletal survey showed 11 ribs on the left but no other evidence of a skeletal dysplasia. An MPS screen was negative. A microarray showed an inherited duplication at 14q32.2 (96,195,807-96,889,316; hg18). A subsequent microarray identified a 200kb deletion at 5q23.1 interpreted as likely benign. He was always macrocephalic (stable) and an overgrowth panel of 11 genes was negative for a pathogenic variant. A duo-exome was performed for this patient and his father that did not identify a cause for his presentation the patient was subsequently enrolled in a research program (Care4Rare Canada).

At 7 years of age, height was 126 cm (+0.3 SD), weight was 29 kg (+1.1), and an OFC was 57.5 cm (+3.8 SD). He appeared macrocephalic with a long face and a prominent chin. He was hyperteloric with long palpebral fissures and lateral eyebrow flare. Ears were small but had normal architecture. He had a bulbous nasal tip, wide nasal base, a smooth philtrum and a thin upper lip. Cheeks were full. Small joints were hyperextensible. He was otherwise proportionate with a straight spine and no pectus. Digits, nails and creases were normal to hands and feet. Given the match through “Genematcher” to *AGO2* observed for another individual recruited to Care4Rare Canada (case 18), the local, Care4Rare database was interrogated for additional variants in *AGO2* and a compelling candidate variant was identified in this individual, Chr8(GRCh37):g.141566342G>A; NM_012154.4:c.1070C>T, p.(Thr357Met). Notably, Sanger sequencing confirmed that the variant was inherited from his mother (case 10). Unfortunately, data on mother´s early developmental milestones were not available. His mother estimates that she walked at around 2 years and her speech was delayed. No clear age for first words and sentences were available. She was held back in school. She took 4 years to complete her final 2 years - but she did graduate. The mother states that, today, she cannot perform simple math (she can only do addition/subtraction under 10), and has a difficult time reading and is easily confused. A formal psychoeducational assessment was never performed. Her mother is deceased and her father is not in contact with the family to provide additional history. She has a full sister with learning difficulties though a clinical assessment and/or carrier testing for the *AGO2* variant has not been performed.

Case 11

A 5-year-old female is the second child to non-consanguineous parents who was naturally conceived. The pregnancy was uneventful. She was born at full term with a birth weight of 4100 grams (+0.2 SD), a birth length of 51 cm (-0.8 SD) and a head circumference of 34 cm (-2.1 SD) with good postnatal weight gain. She was breastfed for six months. The patient was a colicky baby; subsequently, she had

persistent post-feed vomiting and she was diagnosed with gastroesophageal reflux and treated successfully with Nexium. The patient had a seizure at 4 months of age which occurred with concurrent fever a few hours after her first vaccination. This was a prolonged event lasting about 45 minutes and complicated by left-sided weakness. She was commenced on Tegretol and Epilim. She had a further seizure at 6 months after which therapy with Keppra was initiated. She subsequently developed recurrent seizures. Her current EEG shows multifocal spikes on a diffuse background. A cerebral MRI is normal. These have been quiescent singe age 3. Her development is delayed and she has no speech. She is prone to tongue thrusting since shortly after birth. She has periods of intermittent irritability. She sat at about 9 months, mobilised on her knees until age 2.5 years and can now walk, run and jump. She is not waving bye-bye. Some hand stereotypies have been noted as she brings her hands to the midline. She has relatively good eye contact. She has generalized severe eczema, particularly in the flexures which is treated intermittently with wet dressings and regular topical steroids. The patient's head circumference was 49 cm (50th centile). She had a prominent metopic suture, with no other dysmorphisms. She was referred for a genetic consultation for assessment of intellectual disability, autistic features, a prominent metopic suture, epilepsy and behavioural difficulties. Trio-whole exome sequencing analysis detected a heterozygous *de novo* single nucleotide variant *in AGO2*: Chr8(GRCh38)g.140556243G>A; NM_012154.4:c.1070C>T; p.(Thr357Met).

Case 12

This woman is the second child of healthy, non-consanguineous parents with an uneventful family history. The patient was born at 42 weeks of gestation with a normal birth weight. She had a postaxial polydactyly on the right foot, with syndactyly between toes II-II, and IV, V. On the left foot there was syndactyly of toes II-III. Developmental milestones were delayed. From 4 years of age she started to have absences and tonic-clonic seizures that could be controlled by anti-epileptic drugs, but re-appeared after diminishing the medication. There was a behavioral problem in the form of autism and aggressive moods. She has a mild intellectual disability with a total IQ of 58. She can speak in sentences. She had diarrhea in the first years of life that later turned into severe obstipation. An x-rays of hands and feet showed no ossal abnormalities after removal of the extra toe. At 18 years of age hypoplasia of the right kidney with a possible duplex collecting system was detected. At 20 years of age she had a volvulus twice and was found to have a megacolon. Removal of a large part of the bowel was necessary and she got a stoma. She has normal length and head circumference and developed mild obesity in childhood. Brain MRI at 5 years of age showed mildly prominent ventricles and liquor spaces and an abnormal medial temporal collateral sulcus on the left of unclear significance. The cerebellum was normal. Facial dysmorphisms include a high and small nasal bridge, a long nose with a sharp nasal tip, a low hanging columella, short alae nasi, a full lower lip and a short philtrum with hypoplastic maxilla. She has a prominent chin/prognathia with malocclusion of teeth for which she required braces. Her hair is thin. She has mildly dysplastic and small ears. The array-CGH sowed a maternal dupxq24 of 225 kb that is probably benign. Testing of the genes for Smith Magenis syndrome (RAI1) and Smith-Lemli-Opitz syndrome showed normal results. Trio-whole exome sequencing revealed a *de novo AGO2* mutation: Chr8(GRCh37):g.141566321A>G; NM_012154.4:c.1091T>C, p.(Met364Thr).

Case 13

This 6 year old male was born as the first child of healthy non-consanguineous parents. He has an unaffected younger brother and older half-sister. In the family no developmental delays are reported. Labor was induced due to maternal hypertension and the boy was born at 39 weeks of gestation after a cesarean section because of fetal distress. His birth weight was 3452 gram (-0.1 SD). In the neonatal period there were no feeding abnormalities. After the start of solid food he had gag reflexes, which disappeared over time. He started walking independently at the age of 18 months. His further motor development was only slightly delayed. His spoke first word timely, however, further speech

development was severely delayed. Speech therapy was started from the age of two because of drooling. His hearing is normal, except for a slight conductive loss at one ear. At the age of six he can speak sentences. However, because he still has difficulty expressing himself he started to use visual aids to support his verbal communication. A recent formal IQ test resulted in a total IQ score of 55 points, with a disharmonic profile. He has a short attention span and is easily distracted. There are no social interaction or behavioral problems, except that parents report that getting the boy to sleep has been a major struggle for years. On last physical examination his growth parameters were in normal range. He has an epicanthal fold with deep-set eyes. He has open mouth behavior, but normal muscle tone and strength. Metabolic screening revealed no abnormalities. Whole exome sequencing was performed, using a trio approach. This revealed a heterozygous de novo mutation in *AGO2*: Chr8(GRCh37):g.141566321A>G; NM_012154.4:c.1091T>C, p.(Met364Thr).

Case 14

This 6-year old female was born as the second child of healthy non-consanguineous parents. The family history was unremarkable. The pregnancy was complicated by preeclampsia. She was born at 39 weeks of gestation. Her birth weight was 2900 gram (-1.0 SD), birth length was 47 cm (-1.9 SD) and her head circumference was 35.5 cm (+0.7 SD). In infancy she had feeding difficulties, but afterwards at the age of 3 she had transitory hyperphagia. At 5 months of age, she was diagnosed with pulmonary valvular stenosis with an atrial septal defect. Physical examination, at the age of 2.5 months revealed hypertelorism, bilateral strabismus and blue sclera, high-arched palate, slightly more pronounced tongue and an omega-shaped epiglottis. She showed a high tolerance for pain. Her primary and permanent dentition were premature. Due to suspected malabsorption she was on a gluten-free diet until the age of 2 and was also diagnosed with idiopathic hypercalciuria. Both motor and language development were delayed. She walked without support at the age of 23 months, and spoke 10-15 words at the age of 6 years, while she was able to communicate non-verbally. Neurophysiological tests including electromyography and visual evoked potential test were normal. Brain ultrasound revealed bilateral periventricular cysts with frontal horns. Brain MRI revealed slight reduction of white matter. She developed first febrile seizure at the age of 3 years and 3 months. The last examination, at the age of 6 years revealed a friendly child with global developmental delay, proportionately short stature, short neck, abdominal distension, muscular hypotonia and mild obesity. Metabolic screening, karyotype, array-CGH (60K), sub-telomere MLPA (SALSA MLPA P245-B1), DNA-diagnostics for Prader Willi syndrome, Fragile X syndrome, epilepsy gene panels and isoelectric focusing of serum transferrin were all normal. Trio-whole exome sequencing revealed a de novo AGO2 mutation: Chr8(GRCh37):g. 141566329C>G; NM_012054.3:c.1099G>C, p.(Ala367Pro).

Case 15

This 17-year old male is the child of non-consanguineous parents of European ancestry. The family history was unremarkable aside from a paternal male cousin with cerebral palsy. The patient was born at 41 weeks gestation via normal vaginal delivery with a birth weight of 4140 gram (+0.9 SD). Pregnancy was uneventful aside from antenatal diagnosis of bilateral hydronephrosis. APGAR scores were 9 and 10 after 5 and 10 minutes respectively. He had difficulty latching in the newborn period and was diagnosed with torticollis neonatally, requiring physiotherapy. Postnatally, he was followed for right-sided hydronephrosis due to UPJ obstruction. No history of seizures. The patient has high myopia. His growth was normal until 18 months of age, after which the patient had significant weight gain. An endocrinologic etiology for the excessive weight gain was ruled out. Mild delays were noted with gross development. The patient sat independently at 8-9 months of age; and walked at approximately 18 months. His first words were at 12 months of age; however, the patient received speech assessment at 2 years of age due to parental concerns. The patient was diagnosed with autism at age 3. Early childhood assessments noted low average cognitive level. On physical exam at 17years

of age, height was 180 cm (+0.1 SD) and weight was 150kg (+4.1 SD). Previous head circumference measured at age 11 was 57cm (+2SD). On exam, he has deep-set eyes, bifid uvula and hypoplastic tragus. Investigations include normal bone age, Fragile X analysis, cranial MRI, and echocardiogram. Microarray was normal. Trio whole exome sequencing identified a *de novo AGO2* mutation: Chr8(GRCh37):g.141557598C>T NM_012054.3: c.1717G>A; p.(G573S).

Case 16

This 13-year old female was born as the second child of healthy non-consanguineous parents. The family history was unremarkable, except for a mild intellectual disability in a brother of the paternal grandmother. She was born by primary Caesarian section because of a Caesarian section in the maternal medical history. Her development was delayed from the beginning. She learned to walk independently at the age of 27 months and spoke first words between the age of 3 and 4 years. She followed special education and a recent formal IQ test resulted in an IQ score of 47. She had an unilateral myopia of -2 and astigmatism. She wore braces because of malocclusion of her teeth. Her menarche was at the age of 12 years. During puberty she developed temper tantrums. Upon physical examination at the age of 12 years and 9 months she had a height of 159.4 cm (0 SD), obesity with a weight of 76.5 kg (+2.9 SD), and a normal head circumference of 54.,8 cm (+0.5 SD). She had no evident facial dysmorphic features. Her hands and feet were small with a simian crease on the right hand and convex nails. Her skin was soft. In Previous genetic tests, including DNA-diagnostics for Prader Willi syndrome, Fragile X syndrome and myotonic dystrophy, subtelomere MLPA, and genome wide array analysis, were normal. Trio-whole exome sequencing revealed a *de novo* mutation in *AGO2*: Chr8(GRCh37):g.141545641C>G; NM_012154.3:c.2197G>C, p.(Gly733Arg).

Cases 17 and 18 (monozygotic twins)

These are 9-year-old monozygotic female twins born to non-consanguineous parents of Indian descent. The girls were born at 38 weeks gestation by caesarian section due to failure to progress. Case 16's (twin B) birth weight was 2098 grams (-2.6 SD) and birth length was 40.5 cm (-4.3 SD). Case 17's (twin A) birth weight was 2268 grams (-2.1 SD) and birth length was 43 cm (-3.2 SD). Head circumferences at birth are unknown. Case 17 was taken to the NICU due to low birth weight, but both girls had no major neonatal complications and were discharged home with their mother. There were no medical concerns for either case in infancy. Case 17 and case 18 both had a brain MRI at three years of age that was reported as normal. They have also had routine EEGs that were read as normal. Neither girl was known to have seizures. Eye examinations for both girls revealed bilateral exotropia, which required surgery for both girls at 7 years of age. Case 16 also had myopic astigmatism. Hearing for both girls was normal. Both girls were reported to be picky eaters. Case 17 had a history of reflux and case 18 had a history of constipation. Case 17 and 18 were noted to be delayed in their development at 6 months of age. Case 17 started to sit alone at 9 months and walk unassisted at 26.5 months. Case 18 sat alone at 12 months and also walked unassisted at 26.5 months. Both girls spoke first words at about 3 years of age, started to put words together at about 5 years of age, and started to speak in sentences at about 7 years of age. Case 17 and 18 were in special education programs and received therapies since they were toddlers. They have always been progressing, and never regressed. Their cognitive abilities are unknown. At 7 years of age, both girls were unable to read, but they were able to recognize letters. At most recent clinical examination at 8 years of age, facial dysmorphia for both girls included coarse facial features, synophrys, teeth misaligned and yellow, and two large central incisors. Both girls had bilateral clinodactyly and camptodyactyly of the 5th finger. Case 17 had bilateral supernumerary nipples. At 8 years of age, Case 17's weight was 20.4 kg (-1.8 SD), her height was 122 cm (-1.2 SD), and her head circumference was 48 cm (-3.4 SD). Case 18's weight was 22 kg (-1.2 SD), her height was 124 cm (-0.9 SD), and her head circumference was 48.5 cm (-3.0 SD). Genetic testing included normal karyotype, microarray, and metabolic screening. Quad-whole exome sequencing (Case 17 as proband)

identified both girls to be heterozygous for a *de novo AGO2* mutation: Chr8(GRCh37): g.141545586C>T; NM_012154.3: c.2252G>A, p.(Cys751Tyr). Case 17 and case 18 were also found to be compound heterozygous for two variants of uncertain significance in the *SUOX* gene. Biochemical studies gave normal results, indicating that these variants were unlikely to be of clinical significance.

Case 19

This 15-year old girl is the third child of healthy non-consanguineous parents. The family history is unremarkable except for isolated speech delay in a brother. Pregnancy was notable for maternal parvovirus in the third trimester, and subsequent serial ultrasounds. One of which identified an abnormality at the back of the brain. She was born at 37 weeks gestation by vaginal delivery. Birth weight was 2750gram (-1.0 SD), birth length was 46.5 cm (-1.7 SD), and head circumference was 33 cm (-1.0 SD). A postnatal head ultrasound was normal. There were no early infantile issues or hypotonia, however milestones were delayed, and she walked at 16 months. She first came to attention at two years of age as she was not talking. A formal educational assessment at age 3 showed global developmental delay, with most skills below the 1st centile. Her motor issues became less significant throughout childhood; however language and social skills remain poor. Receptive language is better than expressive language. She uses a few words and can put them together, but cannot speak in sentences. She can follow two step commands. She did not meet criteria for autism, but did have parallel play, hand flapping and hand wringing. She is overall friendly. She has obsessive compulsive disorder, issues with skin picking and trichotillomania, and self-injurious behavior. She has significant anxiety, and had an increase in aggression around the time of menarche, which has now resolved. She is unable to print or draw shapes, but can dress independently. She has been healthy, with ongoing symmetric small growth. Significant dental work was required for dental caries, palate expansion, and tooth malignment. Physical examination at age 15 years and 4 months showed small size including height of 148.7cm (-1.96 SD), weight of 41.7kg (-1.7 SD), head circumference of 52cm (-1.93 SD). She had facial dysmorphisms including a slightly asymmetric widow's peak, deep set eyes, upslanting palpebral fissures with hypertelorism, laterally arched eyebrows, a broad-based nose, thin upper lip, wide mouth, chin dimple. Ears have bilateral small and overfolded helices. She has large incisors and teeth appear widely spaced. She required braces and palate expander, multiple cavities. Prior to dental work they were maligned with multiple cavities and protruding from her mouth. There is some prognathism. She has bilateral 5th finger clinodactyly and 4th finger clinodactyly on the right only. Brain MRI had not been done. Genetic testing included a normal microarray and Fragile X testing. Trio whole exome sequencing identified a *de novo AGO2* mutation: Chr8(GRCh37): g.141545586C>T; NM_012154.3: c.2252G>A, p.(Cys751Tyr).

Case 20

This 17-year old male is the second child of healthy non-consanguineous parents. The family history was unremarkable. He was born at 37 weeks gestation with a birth weight of 3084 gram (-1.5 SD), Feeding issues and poor growth started in infancy. He presented at age 20 months with a history of feeding difficulties, failure to thrive, developmental delay, gait abnormality, and dysmorphic features. Bone age was delayed. His height was 75 cm (-2.6 SD), weight 8.6 kg (-2.1 SD) and head circumference 47.5 cm (-0.1 SD). Brain MRI at age 2 years found posterior periventricular and deep white matter signal abnormality in both parietal lobes. He underwent G-tube placement at 2 years of age and started on growth hormone treatment at 4 years of age. At 35 months of age, a developmental evaluation showed cognitive function at 25 months, expressive language at 18-20 months with apraxia, fine motor skills at 18-20 months, and gross motor skills at 28-32 months. He did not speak till age 5 years but was able to communicate through sign. He was treated for precocious puberty at age 9 years. By age 11 he was noted to have intermittent muscle weakness and cramping, hypotonia, with weakness in his hands and ankles with high arched foot, ongoing feeding difficulties, and high arched

palate. Electromyography (EMG) was normal. He will require either surgical intervention or orthodontic braces for his narrow maxillary arch, cross bite, and mandibular jetting. He had perseverative language and developed unusual behaviors in early childhood including: obsessive skin picking and trichotillomania. He was diagnosed with ADD/ADHD, sensory integration disorder, and autism. He developed increasing emotional lability and some aggression with the onset of puberty. He made developmental progress and at age 15 years was working at about the 5th grade level. At age 17 years he is in high school and is classified with mild cognitive impairment. His height was 165.6 cm (-2.6 SD) and weight 65 kg (-2.1 SD). He is social, responds to questions and engages in conversations, makes friends, dances as an extra-curricular activity. He experiences easy fatigability. He has symptoms of narcolepsy and cataplexy (his mother was also diagnosed with narcolepsy). He is being treated by a psychiatrist with good response. He had an extensive genetic evaluation prior to WES with mitochondrial genome testing being sent in 2014. This included panel and targeted gene testing (ATRX, MECP2, Fragile X, CMT panel, Methylation PCR Prader Willi) chromosome analysis and microarray as well as mitochondrial testing (biochemical and MtDNA point mutations/deletions, electron transport chain enzymes). Trio-whole exome sequencing revealed a *de novo AGO2* mutation: Chr8(GRCh37):g.141542706G>C; NM_012154.3: c.2280C>G, p.(Ser760Arg):

Case 21

This 6-year old male was born as the first child of healthy non-consanguineous parents. The family history was unremarkable, except for a mild intellectual disability in two sisters of the mother. Pregnancy was obtained by *in-vitro* fertilization. He was born at 41 weeks of gestation by primary Caesarian section. His birth weight was 3670 gram (-0.2 SD), birth length was 51 cm (-0.9 SD) and his head circumference was 31.5 cm (-3.4 SD). APGAR scores were 9 and 10 after 1 and 5 minutes, respectively. He learned to walk independently at the age of 22 months and presented speech delay. He had school difficulties (help of an educator before 5 years). He had a gastroesophageal reflux in the first months of life. He presented behavioral disorders with intolerance to frustration and limited contact with other children. Physical examination at the age of 5 years 1/2 showed microcephaly with head circumference of 45 cm (-5.0 SD), normal height of 112 cm (+0 SD), and normal weight of 18 kg (-0.5 SD). He had facial dysmorphisms including coarse features, epicanthal fold, anteverted nares, open mouth appearance and full lips. There were no noticeable abnormalities of the extremities. Brain MRI at the age of 3 years was normal. Array CGH identified a deletion including the firsts 3 exons of *AGO2* gene (arr[GRCh37] 8q24.3(141582269_141817600)x1 dn). The deletion occurred *de novo* and was confirmed by qPCR.

Supplementary Methods

Primer list. All primers are shown in 5'-> 3' direction

Primers used for site-directed mutagenesis of human AGO2 expression plasmids

AGO182F	GTGGGCCGCTCCTTCACCGCGTCCGAAG
AGO182R	CTTCGGACGCGGTGAAGGAGCGGCCCAC
AGO192F	GCTCTAACCCTCCTGGCGGGGGCCG
AGO192R	CGGCCCCCGCCAGGAGGGTTAGAGC
AGO201CF	GGGCCGAGAAGTGTGGTTTTGCTTCCATCAGTC
AGO201CR	GACTGATGGAAGCAAAACCACACTTCTCGGCCC
AGOG201VF	GGCCGAGAAGTGTGGTTTGTCTTCCATCAGTCCG
AGOG201VR	CGGACTGATGGAAGACAAACCACACTTCTCGGCC
AGO203F	GGTTTGGCTTCCAACAGTCCGTCCGGC
AGO203R	GCCGGACGGACTGTTGGAAGCCAAACC
AGO357F	CAAAGATGTATTAAAAATTAATGGACAATCAGACCTCAACC
AGO357R	GGTTGAGGTCTGATTGTCCATTAATTTTTAATACATCTTTG
AGO364F	GACAATCAGACCTCAACCACGATCAGAGCGACTGCTAG
AGO364R	CTAGCAGTCGCTCTGATCGTGGTTGAGGTCTGATTGTC
AGO367F	CAACCATGATCAGACCAACTGCTAGGTCGG
AGO367R	CCGACCTAGCAGTTGGTCTGATCATGGTTG
AGO573F	GATCAACGTCAAGCTGGGAAGCGTGAACAACATCCTGCTG
AGO573R	CAGCAGGATGTTGTTCACGCTTCCCAGCTTGACGTTGATC
AGO733F	TGGAAACATTCCAGCACGCACGACTGTGGAC
AGO733R	GTCCACAGTCGTGCGTGCTGGAATGTTTCCA
AGO751F	GTTCGACTTCTACCTGTATAGTCACGCTGGCATC
AGO751R	GATGCCAGCGTGACTATACAGGTAGAAGTCGAAC
AGO760F	GCTGGCATCCAGGGGACAAGGAGGCCTTCGCACTATC
AGO760R	GATAGTGCGAAGGCCTCCTTGTCCCCTGGATGCCAGC

Primers used for PCR amplification/Sanger sequencing of AGO2 variants

4F	ACCTGTGCGGTGTCTTTAAACGTC
4R	ACCCAACACTGCAGGTGAGAC
5F	TCACAGTGTCCCACACAGCA
5R	GCCGTAACCCACCCAACACT
9F	GCGTGACGTGGGGTTTACTTAACA
9R	TCCGTTCCAAGCATCAGAGGTTTC

Primers used for qPCR analysis of patient 21

SULF1; 8q13.2-13.3

SULF1 for	CCCCCAAGAAATGGTCACTA
SULF1 rev	CAGGCAAGACTGCCCTAGAC

PTK2 exon 22; 8q24.3

PTK2_22 For	TGGGAGATACTGATGCATGG
PTK2_22 Rev	GGTCATAGGCCCAGCATTTC

PTK2 exon 32; 8q24.3

PTK2_32 For	TGGGTGAGCTCATCAACAAG
PTK2_32 Rev	GCCCAAGCATTTTCAGTCTT

AGO2; 8q24.3

AGO2 For	GATATGCCTTCAAGCCTCCA
AGO2 Rev	AACTCTCCTCGGGCACTTCT

Non-biased molecular dynamics (MD) simulations. We simulated wild-type (WT) AGO2 and eleven variants (Figure 1a). To capture possible effects of the variants along the RISC pathway, we simulated

WT and mutant AGO2 in apo, guide-bound (core-RISC) and guide•target-bound (holo-RISC) states (Figure S13). Since the crystal structure of the free AGO2 is not available, after removing all RNA atoms, we used a duplex-bound structure (PDB ID: 4W5T) for the apo-AGO2 simulations. For the core-RISC, we ran two independent sets of simulations starting from X-ray structures with different sequences of the miRNA guide, with PDB ID 4OLA and 4W5N. The most striking difference between these structures is in their g7 nucleotide. While in the 4OLA structure, I365 intercalates between A6 and A7, forming a kink [1], in the 4W5N structure U7 is shifted away from I365[2] (Figure S12). The sets derived from the 4OLA and 4W5N structures are denoted below as intercalating (*int)* and non-intercalating (*nonint*) core-RISC states, respectively. For the holo-RISC state we also ran two independent sets of trajectories (Figure S12): with a target fully matched to the guide seed (g2-8, 4W5Q) and with a U•C mismatch at g8 position (g2-7, 4W5T). In total, we ran 60 independent non-biased MD trajectories (for each, WT and all 11 variants, for each of the five states).

Since in all X-ray structures some protein residues were not resolved, these parts were manually added to the models using Psfgen plugin of the VMD suite[3]. Missing RNA residues in the RNA-bound states were added the same way as missing protein residues. Mg^{2+} ions from the X-ray structures remained in the models, while all phenol molecules were deleted. We used CHARMM36 force field in all MD simulations[4]. Particle Mesh Ewald method[5] was applied to treat electrostatic interactions and a cutoff of 12 Å was used for the van der Waals interactions. NAMD 2.12 package was used for all MD simulations[6].

All manually added protein and RNA residues were optimized for 10,000 steps of steepest-descent algorithm while the rest of the structure was restrained with a force constant of 100 kcal/mol/Å^2. Thereafter, the structures were solvated in ~ 120×120×120 Å TIP3P water box[7] and Na^+ and Cl^- ions were added to neutralize the systems and establish a biologically relevant 150 mM concentration of NaCl. The resulting structures were subjected to 1,000 steps of steepest-descent optimization of water and ions coordinates while the rest of the structure was restrained with a force constant of 50 kcal/mol/Å^2, following the 1 ns of NVT simulations with 1 fs time-step at 298K using the same restraints. The obtained coordinates were used for 10,000 steps of steepest-descent optimization without any restraints. The optimized coordinates were used for 400 ps of gradual heating of the systems to 298 K with 1 K increment every 400 fs. The structures were subjected to 1-2 ns of NPT equilibration with a 1 fs time-step at standard conditions using Langevin thermostat and barostat and the cell volume equilibration was verified. Afterwards, we introduced the studied mutations into the protein structure using Mutator plugin for VMD[3]. The F182del mutation was introduced by manually deleting coordinates of the F182 residue. Each mutated structure was subjected to 10,000 steps of steepest-descent optimization of the mutated residue and two of its neighboring residues, while the rest of the structure was restrained with 30 kcal/mol/Å^2. In the case of the F182del mutation, six residues neighboring the deletion site were subjected to optimization. Obtained WT structures and partially optimized structures of the mutated protein were used for NVT simulations at standard conditions with 2 fs time-step using SHAKE/RATTLE algorithm and coordinates saved each 4 ps. Each trajectory length was at least 200 ns in apo and core-RISC states, or at least 400 ns in holo-RISC states, resulting in 60 trajectories with a cumulative simulation time of app. 17 µs.

Initial structures of the WT with compromised guide 3′-end anchoring in the *int* core-RISC and g2-7 holo-RISC states were prepared manually by changing coordinates of g18-g21 residues in WT frames from the corresponding trajectories to aligned coordinates of unbound g18-g21 residues in *nonint* core-RISC M364T and g2-7 holo-RISC L192P trajectories, respectively. After a restrained optimization of the moved g18-g21 residues and surrounding atoms, we started NVT simulations from these initial structures (denoted as WTΔ(3′-PAZ)) as described above.

Principal component analysis. Principle component analysis (PCA) was used to find the largest amplitude modes within the MD trajectories and initial X-ray structures. Prior to analysis of the MD trajectories, we used PCA to extract the largest modes of variance in the X-ray structures of hAgo2. We applied PCA to the set of all available X-ray structures of WT AGO2 at the time of the study (n = 19)

based on the coordinates of Cα atoms of the largest common set of protein residues in those structures (*m* = 3 Cartesian coordinates × 764 res. out of 859 res. in the full AGO2 protein). All X-ray structures were aligned to 4W5T structure based on *m*, and covariance of each coordinate was calculated. Diagonalization of *m*×*m* covariance matrix provided a set of its eigenvectors (principal components, PC), arranged in descending order of their corresponding eigenvalues, which reflect the magnitude of variance along their vectors.

The same set of residues from the X-ray structures was also used for PCA of all MD trajectories in the free state. Trajectories were aligned to their corresponding initial X-ray structures based on *m*, followed by the above described procedure of PCA. Projections on PCs were calculated as root mean square deviations (RMSD) along the PC vectors using 4W5T structure as a reference. Contributions of the protein residues to PC1 were estimated by calculating vector lengths from three Cartesian components at each residue. ProDy1.9 package was used for PCA calculations[8].

Metadynamics (MetD). MetD is an enhanced sampling method widely used in molecular modeling to reconstruct free energy profiles by periodically adding positive Gaussian potentials to a potential energy function of selected degrees of freedom, thus allowing a system to escape local minima[9]. These degrees of freedom are called collective variables (colvars) and used to describe a simulated process. Two non-tempered MetD runs (Gaussian height was constant) were performed in g2-7 holo-RISC: WT and L192P. Both initial structures were taken from the random frames of the equilibrated parts (t>150 ns) of the corresponding non-biased trajectories. In these runs we used I365δ-g(6-7) as a colvar (see Supplemental Figure 14a). The width and height of the Gaussians and their deposition rate were 0.1 Å, 0.05 kcal/mol and 2 ps^{-1}, respectively. All other MD parameters were set as in the non-biased NVT simulations described above. Both WT and L192P MetD trajectories were simulated for app. 360 ns each and denoted as 1D MetD. The grid for the hill deposition (1.5 Å, 8 Å) was narrower than the actual range of sampling along this colvar during simulations. Therefore, the obtained PMF cannot be used and was thus not presented. Colvar module of NAMD was used for MetD simulations and free energy profiles calculations[10].

Analysis and visualization. VMD 1.9 package[3] was used to parse the MD trajectories, and to produce the molecular graphics images and movies with the Tachyon renderer, using also the MSMS plugin to visualize molecular surfaces[11].

Supplementary Figures

AGO2	F182	L192	G201, H203	T357, M364, A367	G573	G733	C751	S760
Homo sapiens	GRSFFTAS	SNPLGGG	VWFGFHQSV	KKLTDNQTSTMIRATAR	KLGGVNN	IPAGTTV	FYLCSHA	QGTSRPS
Macaca mulatta	GRSFFTAS	SNPLGGG	VWFGFHQSV	KKLTDNQTSTMIRATAR	KLGGVNN	IPAGTTV	FYLCSHA	QGTSRPS
Felis catus	GRSFFTAS	SNPLGGG	VWFGFHQSV	KKLTDNQTSTMIRATAR	KLGGVNN	IPAGTTV	FYLCSHA	QGTSRPS
Gallus gallus	GRSFFTAS	SNPLGGG	VWFGFHQSV	KKLTDNQTSTMIRATAR	KLGGVNN	IPAGTTV	FYLCSHA	QGTSRPS
Mus musculus	GRSFFTAS	SNPLGGG	VWFGFHQSV	KKLTDNQTSTMIRATAR	KLGGVNN	IPAGTTV	FYLCSHA	QGTSRPS
Xenopus laevis	GRSFFTAS	ANPLGGG	VWFGFHQSV	KKLTDNQTSTMIRATAR	KLGGVNN	IPAGTTV	FYLCSHA	QGTSRPS
Danio rerio	GRSFFTPS	SNPLGGG	VWFGFHQSV	KKLTDNQTSTMIRATAR	KLGGVNN	IPAGTTV	FYLCSHA	QGTSRPS
Homo sapiens AGO1	GRSFFSPP	YHPLGGG	VWFGFHQSV	KKLTDNQTSTMIKATAR	KLGGINN	IPAGTTV	FYLCSHA	QGTSRPS
Homo sapiens AGO3	GRSFFSAP	DHPLGGG	VWFGFHQSV	KKLTDNQTSTMIKATAR	KLGGINN	IPAGTTV	FYLCSHA	QGTSRPS
Homo sapiens AGO4	GRSFFSPP	YHPLGGG	VWFGFHQSV	KKLTDNQTSTMIKATAR	KLGGINN	VPAGTTV	FYLCSHA	QGTSRPS

Supplementary Figure 1. Patient-mutations affect highly conserved residues. All amino acids mutated in patients, each marked in red, are evolutionary highly conserved from humans to zebrafish. Additionally, they are also highly conserved in other Argonaute proteins AGO1, 3 and 4. Amino acids that are not conserved are indicated in blue.

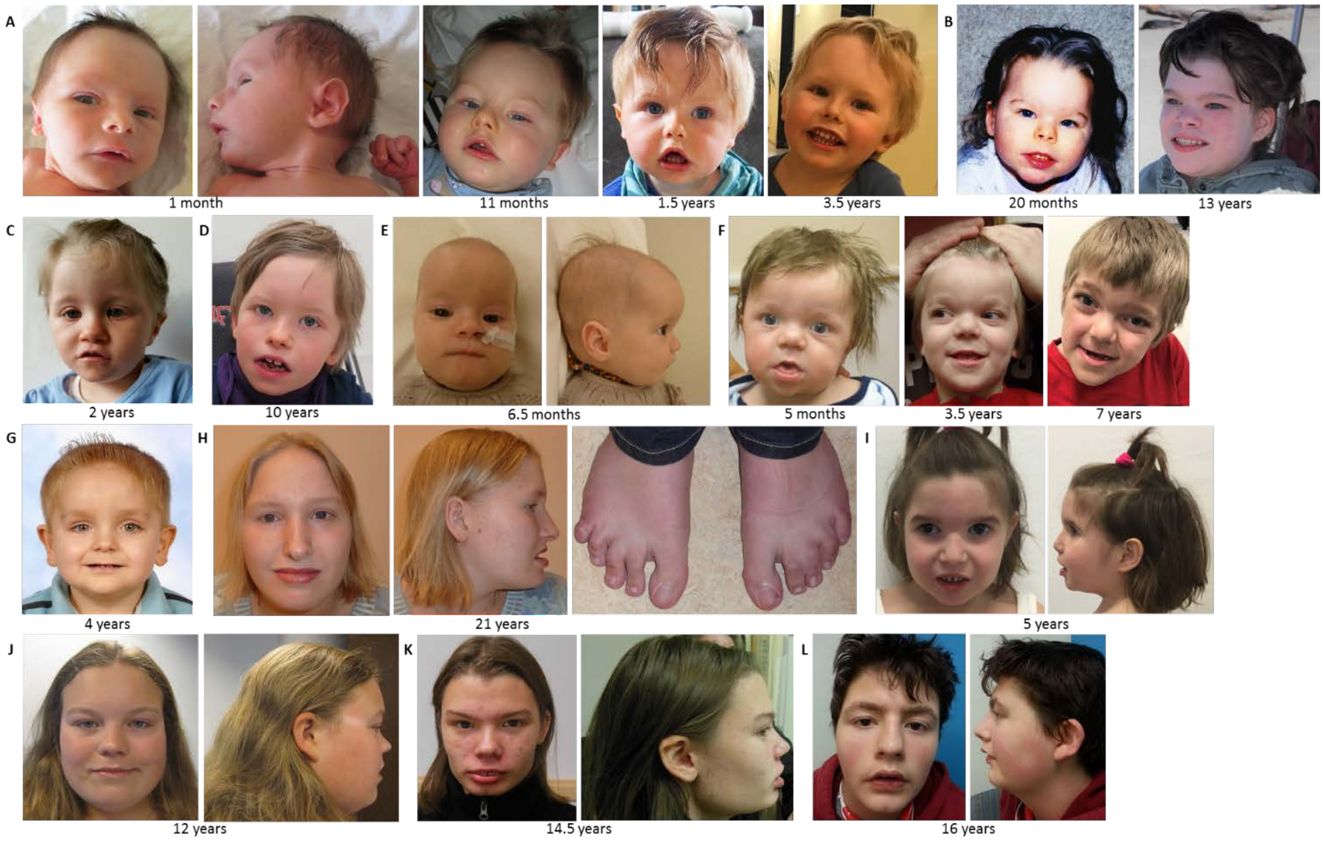

Supplementary Figure 2. Facial phenotype of individuals affected by the AGO2 associated disorder. **a.** Facial images of case 2 at the ages 1 month, 11 months, 1.5 and 3 years. **b.** Facial images of case 3 at 20 months and 13 years. **c.** Facial image of case 4 at 2 years. **d.** Facial image of case 5 at 10 years. **e.** Facial image of case 6 at 6.5 months. **f.** Facial images of case 9 at 5 months, 3.5 and 7 years. **g.** Facial image of case 7 at 4 years. **h.** Facial and feet images of case 12 at 21 years. Note syndactyly at toes II-III. **i.** Facial images of case 14 at 5 years. **j.** Facial images of case 16 at 12 years. **k.** Facial images of case 19 at 15 years. **l.** Facial images of case 20 at 16 years.

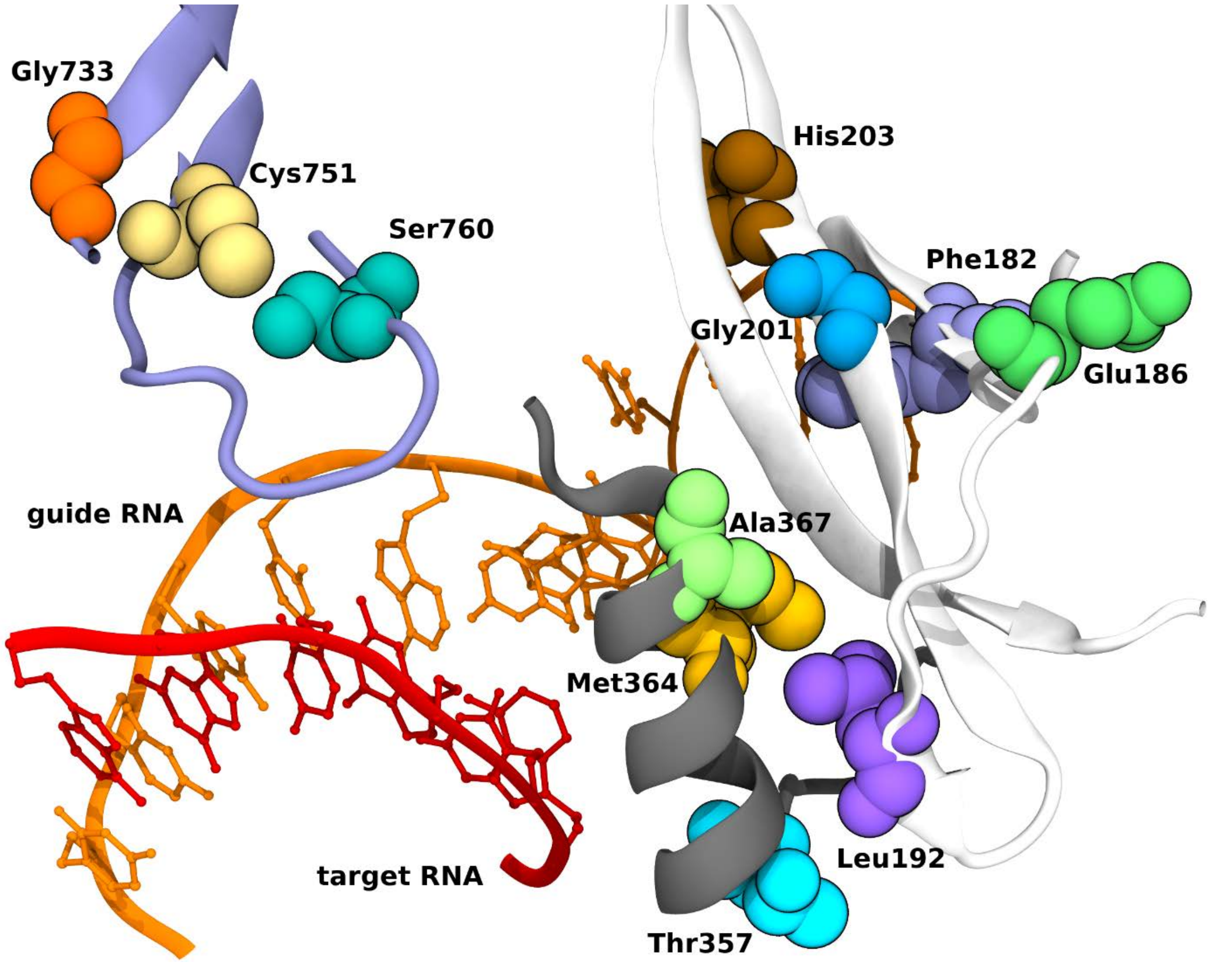

Supplementary Figure 3. Positions of amino acids in AGO2 mutated in affected individuals. This view is based on the structure of AGO2 in complex with a guide and a target RNA, shown in blue and orange respectively; accession 4w5t *(21)*. Note that mutated residues cluster in space at three positions: at the helix7/L1 interface (Leu192; Thr357; Met364 and Ala367), at the upper end of L1 (Phe182; Gly201 and His203), and close to a loop which contacts the minor groove of the RNA double helix (Gly733; C751 and Ser760).

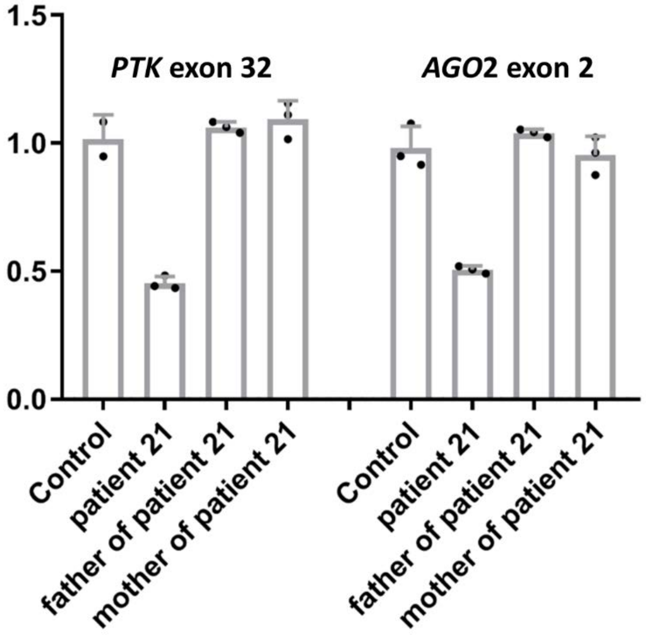

Supplementary Figure 4. *AGO2* and *PTK2* DNA copy number analysis in case 21 and his parents. qPCR analysis of the 235.3-kb *de novo* deletion identified in case 21 (chr8:g.(141569559_141582269)_(141817600_141829173)del, GRCh37,NC_000008.10). Shown are *AGO2* and *PTK2* exonic copy number values relative to *SULF1* in case 21, his both parents and one independent unaffected individual (n=2-3 biologically independent experiments). Data are presented as mean values + SD. Source data are provided as a Source Data file.

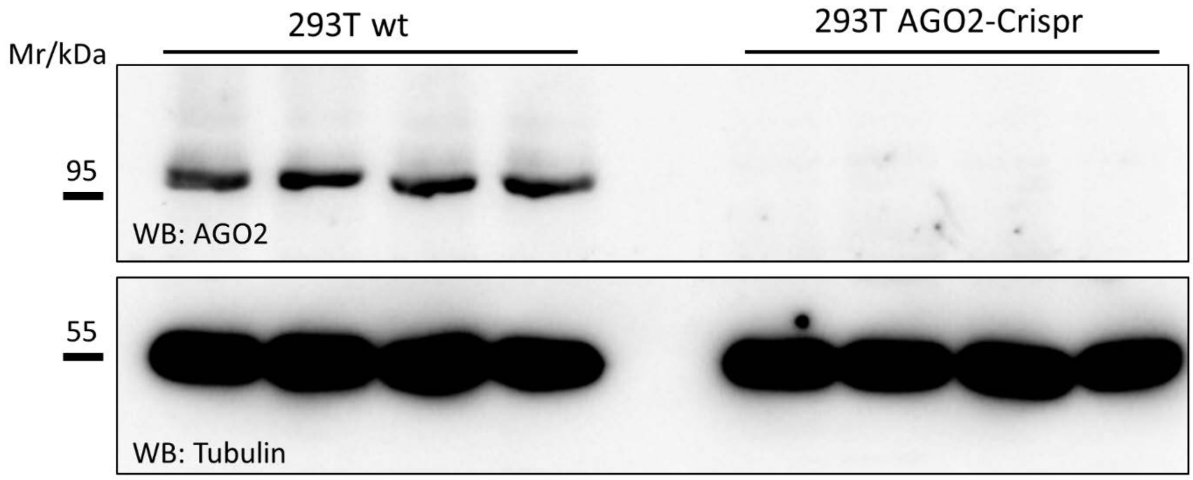

Supplementary Figure 5. Generation of an AGO2 deficient HEK293T cell line. An AGO2 deficient cell line was generated using Crispr/Cas9 technology from the HEK293T parental cell line. Cell lysate samples (four samples each) were analyzed by Western blotting using antibodies against AGO2 and α-tubulin. Note the complete absence of AGO2 in AGO2-Crispr cells. Rat anti-AGO2 and mouse anti-tubulin antibodies were used at 1:1000 dilution. Source data are provided as a Source Data file.

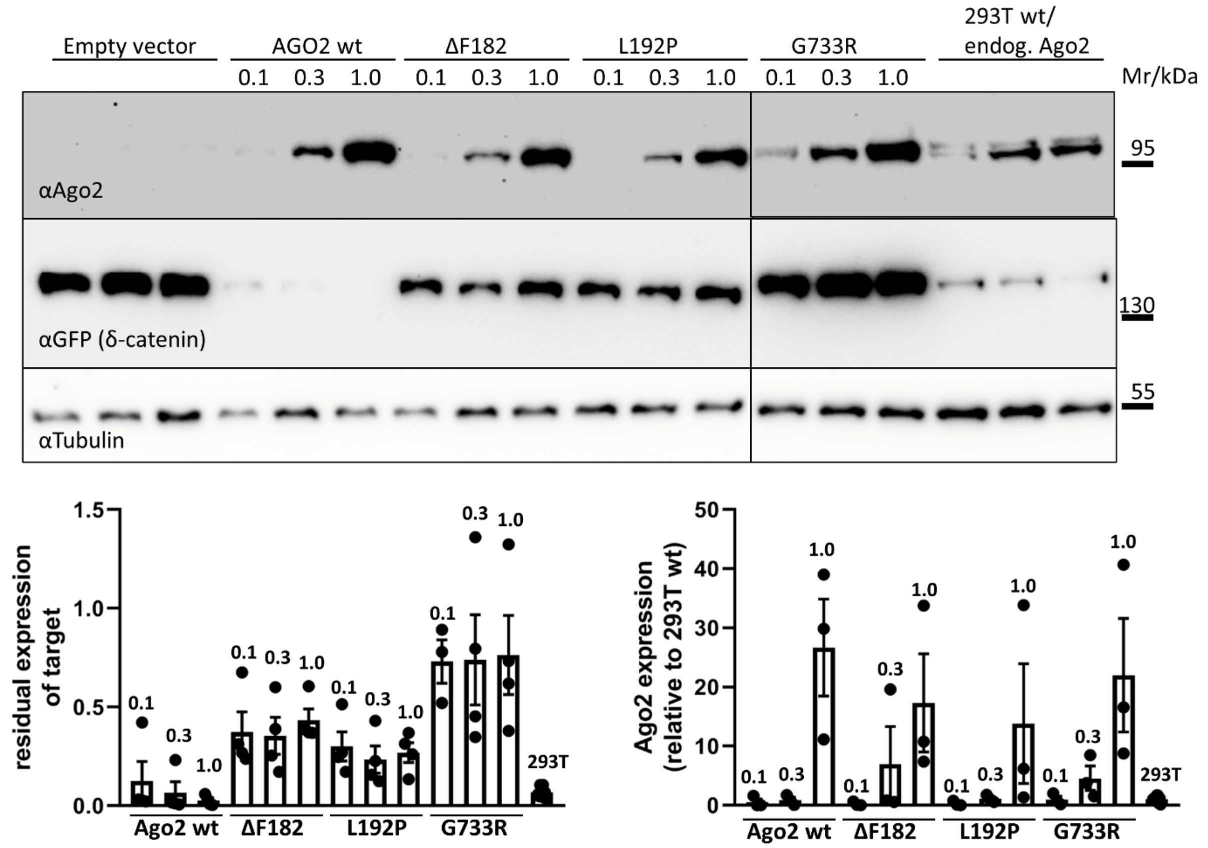

Supplementary Figure 6. shRNA silencing by AGO2 variants at different expression levels. AGO2-deficient, or non-modified HEK293T wt cells were plated next to each other in 12-well plates. For transfection, a master silencing mix containing expression vectors for mRFP, GFP-δ-catenin and the shRNA construct for δ-catenin was prepared. After distribution to individual tubes, different amounts (0.1 µg/0.3 µg/1.0 µg) of expression vectors for Flag/HA-tagged AGO2 variants were added, and empty Flag/HA-expression vector was added to a total amount of 1.0 µg for each tube. Control AGO2-deficient cells, as well as HEK293T wt cells, received only silencing mix and 1 µg of empty Flag/HA-vector. Plasmid mixes were transfected into cells using Turbofect reagent. On the next day, efficient transfection was verified by monitoring mRFP fluorescence on a fluorescence microscope (only wells with >70 % of cells transfected were included). Cells were lysed and analysed by Western blotting, using the antibodies indicated. The experiment was repeated four times with similar results. For quantifications, the intensity of GFP-δ-catenin signal was divided by the average signal obtained from the three “empty vector” wells (left bar graph; n=4 biologically independent experiments). For determining the relative amounts of AGO2 expression, the signal obtained with the AGO2 antibody was first divided by the tubulin signal in the same lane. These numbers were then divided by numbers obtained with HEK293T wt cells (right bar graph; n=3 biologically independent experiments). Note that AGO2 wt, even at the low expression level where it is hardly detectable in the Western Blot (0.1 µg of DNA; 54 % of AGO2 protein expression in HEK293T wt cells), silences more efficiently than F182del, L192P and G733R mutant forms of AGO2 at 10- to 20-fold overexpression. Data are presented as mean values +/- SEM. Source data are provided as a Source Data file.

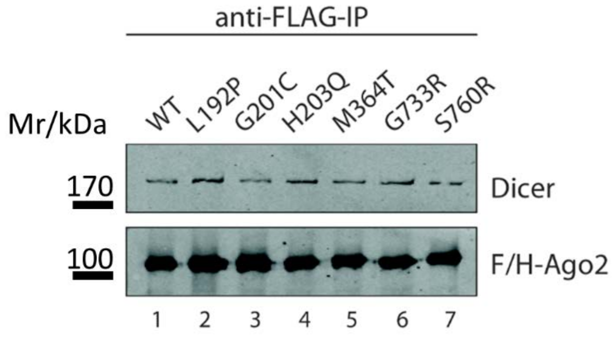

Supplementary Figure 7. Interaction of AGO2 mutants with Dicer. Flag/HA-tagged (F/H-) AGO2-WT and mutant variants were overexpressed in HEK293T cells, immunopurified from cell lysates by anti-FLAG-IP, separated by SDS-PAGE, and analyzed by Western blotting. Co-precipitated Dicer was detected by anti-Dicer antibody. Flag/HA-tagged AGO2 variants were detected by anti-HA antibody. The experiment was repeated independently three times with similar results. Source data are provided as a Source Data file.

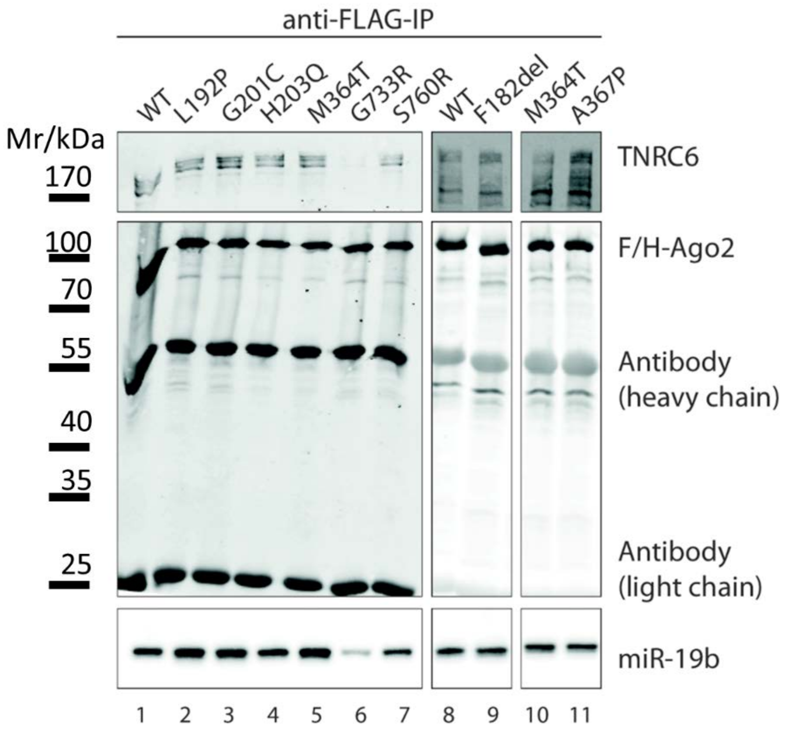

Supplementary Figure 8. **Interaction of AGO2 mutants with TNRC6 and miR-19b.** Flag/HA-tagged AGO2-WT and mutant variants were overexpressed in HEK293T cells, immunopurified with anti-FLAG-IP, and analyzed by Western blotting. Co-immunoprecipitated TNRC6 proteins were detected by pan-TNRC6-antibody, clone 7A9 (Millipore). Flag/HA-tagged AGO2 variants were detected by anti-HA antibody. Co-precipitated miR-19b was detected by Northern Blotting. The experiment was repeated independently three times with similar results (twice for F182del/lane 9 and A367P/lane 11). Source data are provided as a Source Data file.

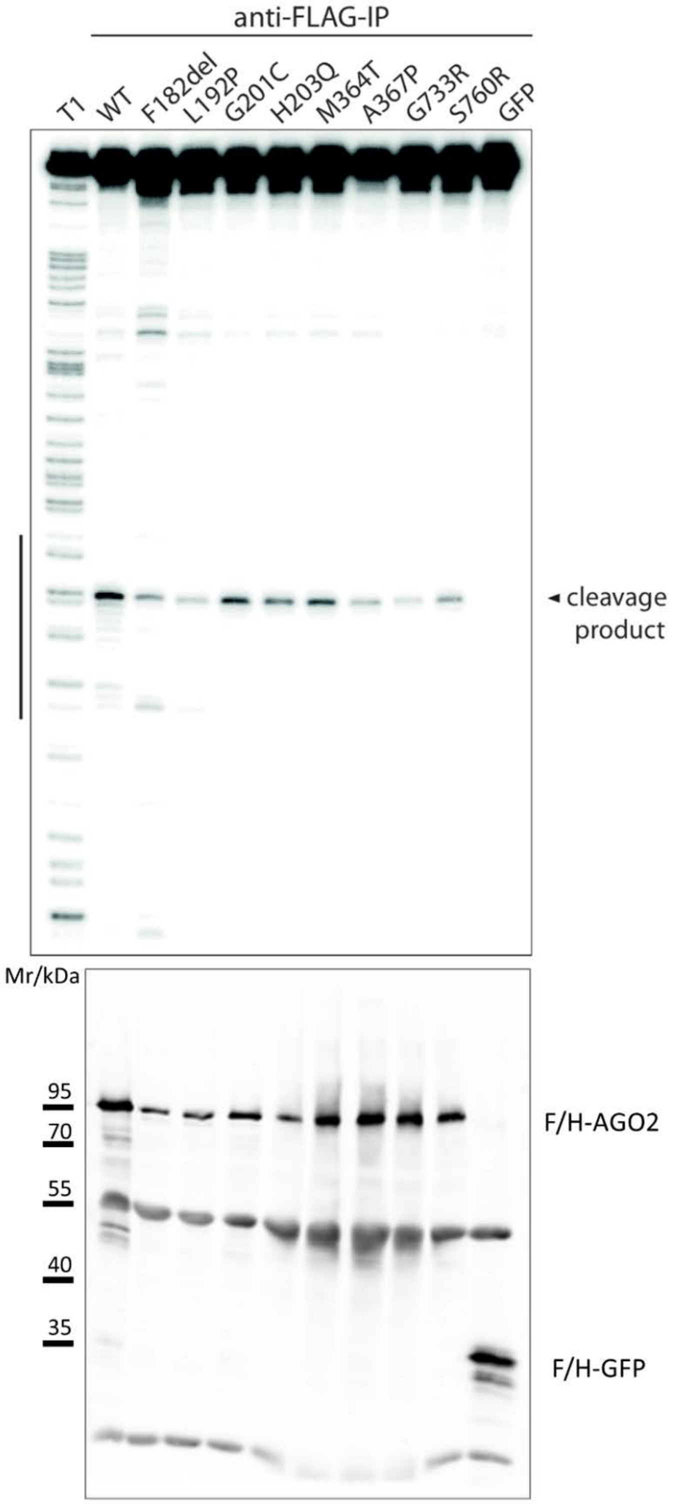

Supplementary Figure 9. *In vitro* cleavage assay of AGO2 mutants. F/H-tagged AGO2-WT and mutant variants overexpressed in HEK293T cells were immunoprecipitated. Precipitates were incubated with a radiolabeled RNA substrate fully complementary to the endogenous miR-19b. A partial digest of the radiolabeled substrate by RNase T1 is indicated by 'T1'. Samples were analyzed by gel electrophoresis, followed by autoradiography (upper panel). The region complementary to miR-19b is shown by a vertical black bar. An aliquot of the immunoprecipitated protein was analyzed by Western blot using anti-HA antibody (lower panel). The experiment was repeated independently three times with similar results (twice for F182del and A367P).

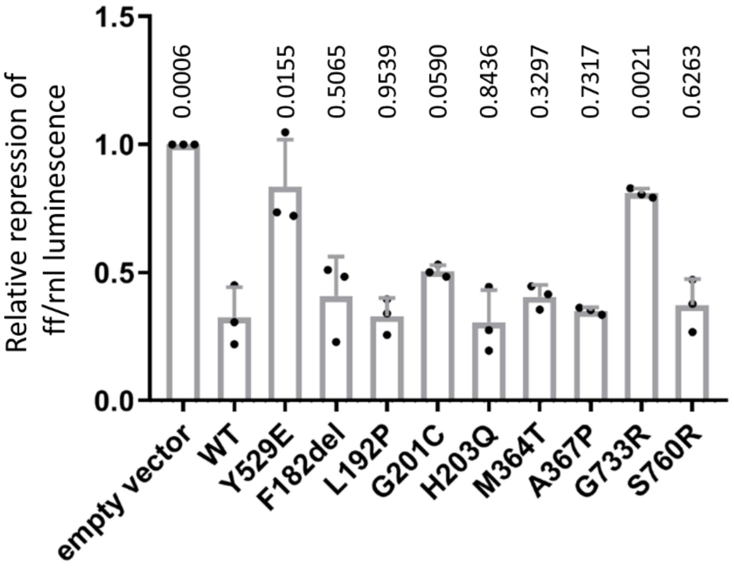

Supplementary Figure 10. Luciferase reporter assay of AGO2 WT and mutants. The dual luciferase reporter plasmid pMIR-HMGA2, containing the 3′-UTR of HMGA2, was transfected with F/H-Ago2 expression constructs, into Hela cells. Dual luciferase assays were performed to observe the repression by overexpressed F/H-Ago2 and mutants. (replicates ≥ 3; p-values were calculated using a two-tailed Student's t-test in relation to WT, exact p-values are given). Source data are provided as a Source Data file.

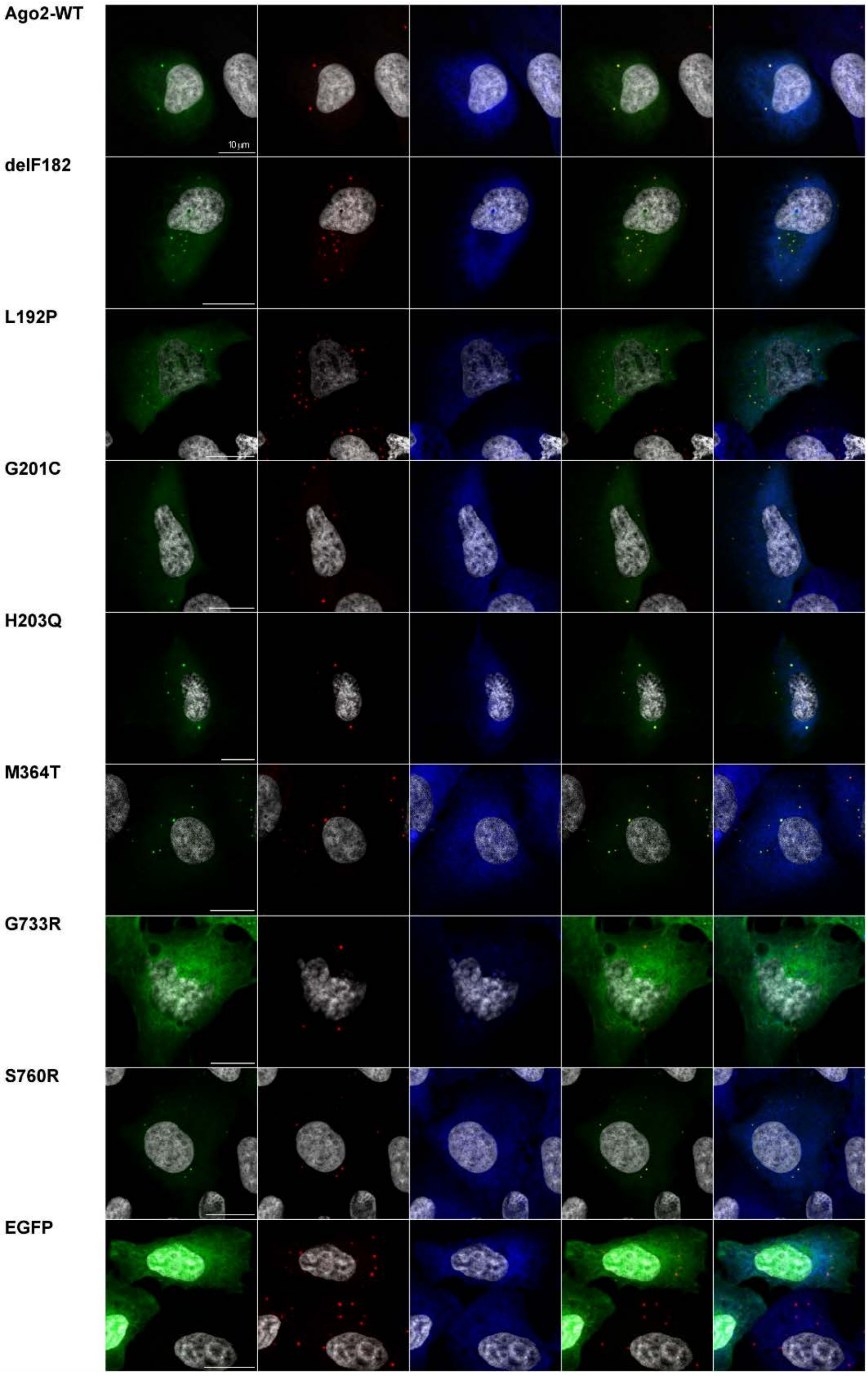

Supplementary Figure 11. Cellular localization of AGO2 mutants. Immunocytochemical detection of AGO2-GFP fusion proteins (GFP, green), DDX6 (red, P-body marker) and endogenous ATXN2 (blue, endogenous stress granule marker) in transfected U2OS cells. Note that AGO2-WT and most of the mutant variants localized in cytoplasmic puncta which are confirmed to be P-bodies by co-staining with DDX6. Only the G733R variant is diffusely localized in the cytoplasm, similar to EGFP alone. Each variant was examined in at least three independent biological replicates. For each replicate, more than 30 cells were visually analysed. Scale bar: 10 µm.

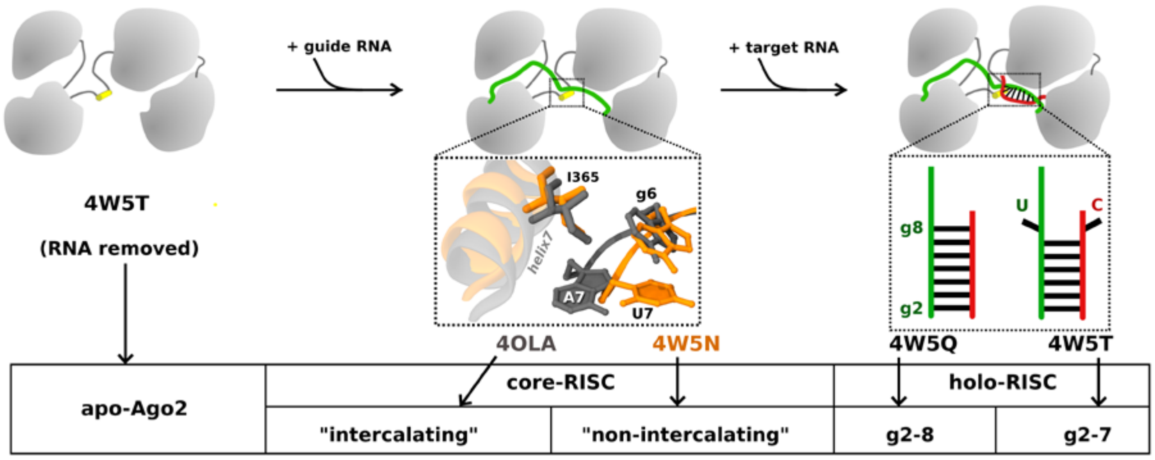

Supplementary Figure 12. Simulated complexes. WT and all AGO2 variants were simulated in five states of the AGO2-RNA complex: apo-Ago2, two states of the AGO2•guide complex (core-RISC) and two states of the hAGO2•guide•target complex (holo-RISC). The reference structures of the two core-RISC states (4OLA and 4W5N) differ by the helix7-guide interactions and are designated as "intercalating" (*int*) and "non-intercalating" (*nonint*) states, respectively. The two holo-RISC states (4W5Q and 4W5T) contain fully complementary guide-target duplex or exhibit a mismatch at g8, and denoted as g2-8 and g2-7, respectively.

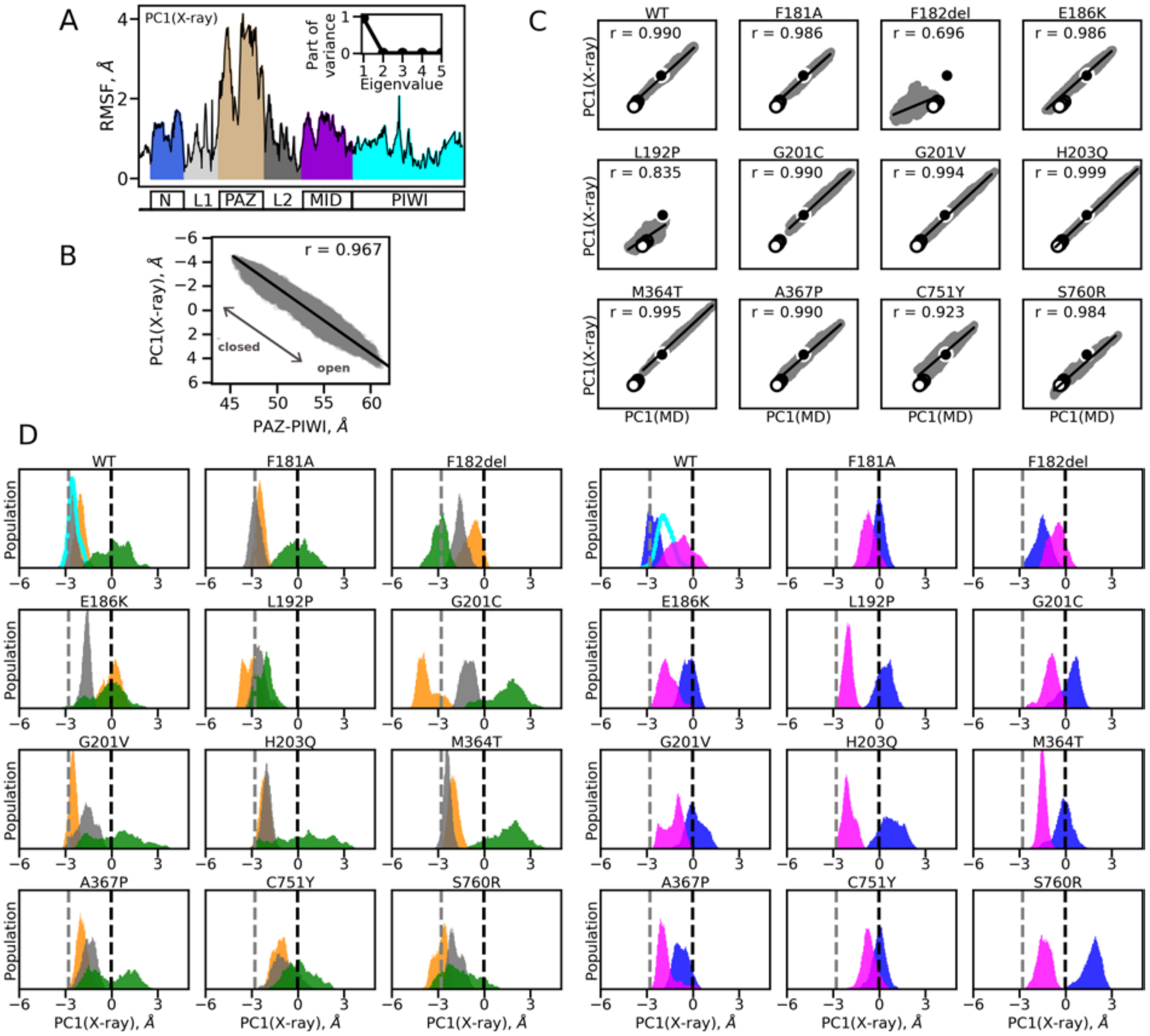

Supplementary Figure 13 Open-closed mode in AGO2. **a.** Per-residue root mean square fluctuation (RMSF) of PC1 from the set of AGO2 X-ray structures (PC1(X-ray)). Inset, the fraction of total variance in the set of AGO2 X-ray structures explained by the first five eigenvalues of the covariance matrix in the PCA. **b.** Linear correlation between the projection of concatenated apo-AGO2 trajectories of all variants on PC1(X-ray) and the center of mass (c.o.m.) PAZ-PIWI distance. r, Pearson's correlation coefficient. This high correlation demonstrates that PC1(X-ray) is in fact the open-closed conformational mode. **c.** Linear correlations between the projections of apo-AGO2 trajectories of each variant on their own corresponding PC1 (PC1(MD)) and on PC1(X-ray); black and white circles denote projections of the duplex-bound and the guide-bound WT AGO2 X-ray structures on the two vectors, respectively. r, Pearson's correlation coefficients. For WT-AGO2 and all variants, except F182del and L192P, PC1(MD) highly correlated with PC1(X-ray) implying that the dominant conformational mode of the protein in the corresponding trajectories is the open-closed mode. **d.** Distributions of non-biased trajectories on the open-closed mode. The population distributions were calculated from the last 100 ns of each trajectory. Color code of the states: apo-AGO2 – green, *int* core-RISC – gray, *nonint* core-RISC – orange, g2-8 holo-RISC – magenta and g2-7 holo-RISC – blue. Cyan curves in the WT plots denote the WT with the manually removed 3′-end of the guide from its binding site at the PAZ domain (WTΔ(3′-PAZ)) in the int core-RISC state (left) and g2-7 holo-RISC state (right). Populations of the projections of all trajectories on PC1(X-ray) were normalized to the same bin number (60). Black and gray dashed lines denote projections of the duplex-bound and the guide-bound WT AGO2 X-ray structures on PC1(X-ray), respectively.

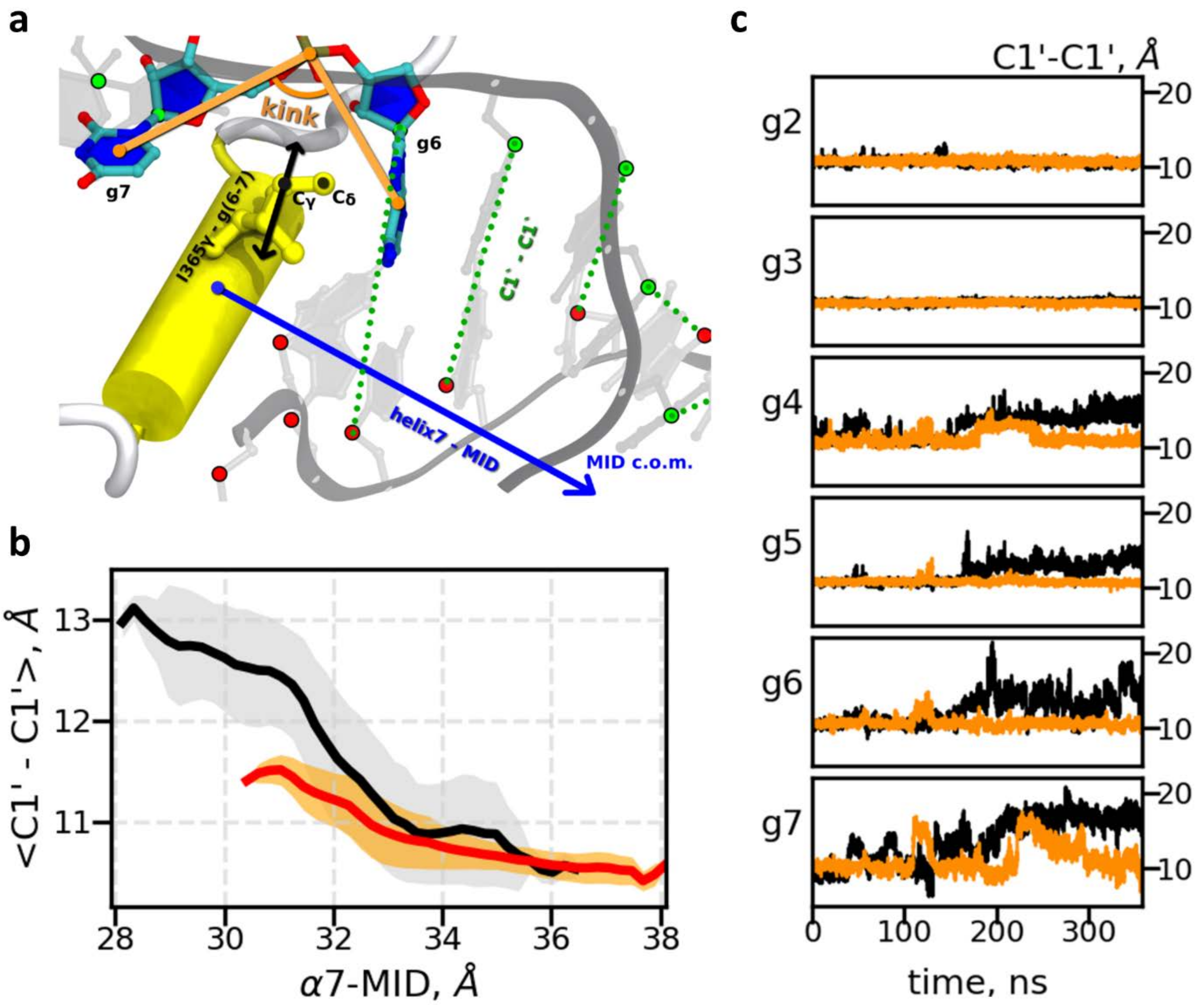

Supplementary Figure 14. a. View of helix 7 of AGO2 in close contact to a guide-target double helix. Collective variables and non-biased structural variables used in simulations are indicated. Blue and red circles denote the C1′ atoms of the guide and the target RNA, respectively. The distance between the Cδ atom of I365 and the center of mass (c.o.m.) of g6 and g7 nucleobases defines the intercalation of I365 between base pairs. The kink between g6 and g7 is induced by enhanced sampling of I365 intercalation between g6 and g7 (Figure S15). **b.** Guide-target duplex width (calculated as a mean base pair width of five base pairs at g2 to g6, <C1′-C1′>) as a function of helix-7-MID distance (α7-MID), determined from metadynamics trajectories of WT and p.L192P. The curves show the mean value with standard deviation as shaded areas. **c.** Individual trajectories for all C1′-C1′ distances at positions g2 to g7 from 1D MetD; black and orange colors denote WT-AGO2 and L192P, respectively.

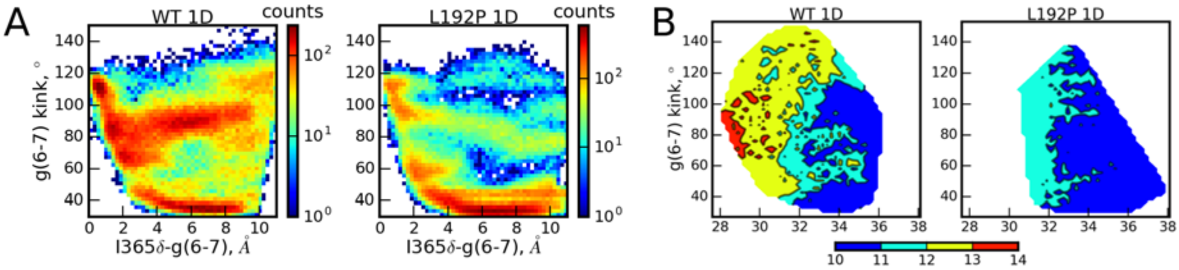

Supplementary Figure 15. **Intercalation-induced structural changes in the MetD trajectories. a**. Intercalation of I365 between the g6 and g7 nucleotides of the guide induces a kink between them. The kink angle is measured as c.o.m.(g6 base)-P(g6)-c.o.m.(g7 base). **b**. Guide-target duplex width (calculated as a mean base pair width of five basepairs at g2 to g6, <C1'-C1'>) as a function of helix7-MID c.o.m. distance (α7-MID) and the g6-g7 kink angle.

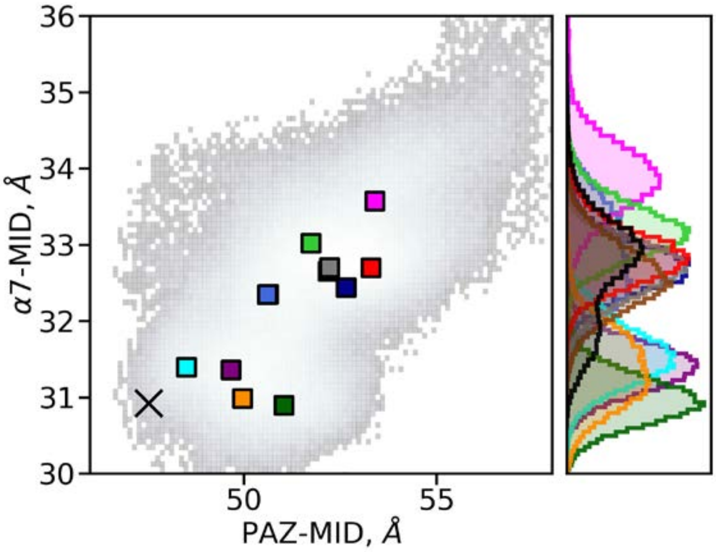

Supplementary Figure 16. **Population density maxima of the trajectories in the g2-8 holo-RISC state.** Density maxima are calculated from their last 100 ns. For more details see Fig. 4d captions. The difference between the population distribution in g2-8 and g2-7 states (Fig. 4d) can potentially be attributed to the different RNA sequences.

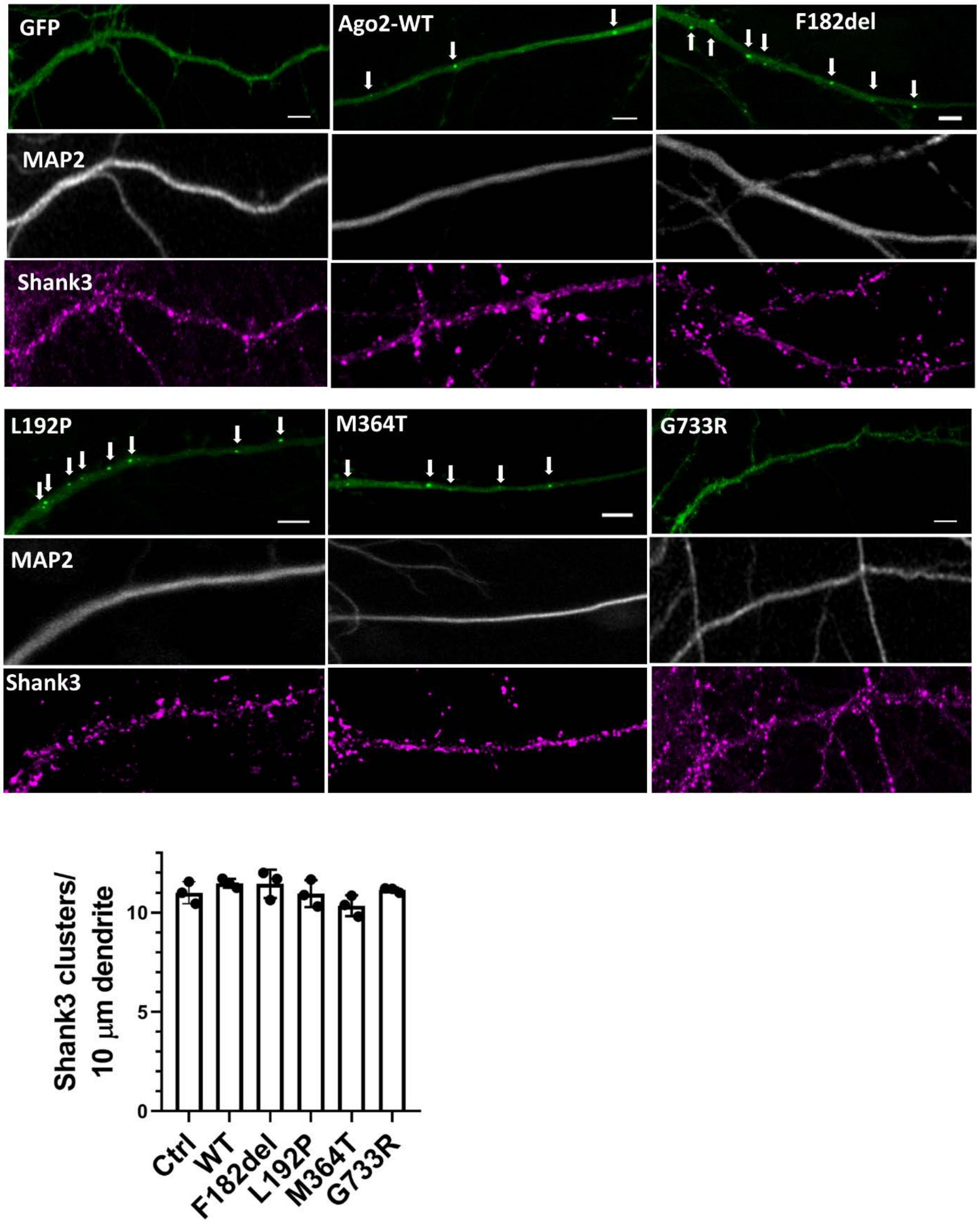

Supplementary Figure 17. Expression of AGO2-WT or mutant does not alter the density of postsynaptic Shank3 clusters on dendrites. Primary cultured hippocampal neurons were transfected as in Fig. 4A; in addition to the GFP signal and MAP2 staining, staining for endogenous Shank3 is shown. The density of dendritic Shank3 clusters is represented as a bar graph (mean +/- SD; n=3 biologically independent experiments, with 9-17 cells evaluated per experiment and per experimental condition). Source data are provided as a Source Data file. Scale bar, 5 µm.

Supplementary references.

1 Schirle, N. T. & MacRae, I. J. The crystal structure of human Argonaute2. *Science* **336**, 1037-1040, doi:10.1126/science.1221551 (2012).

2 Schirle, N. T., Sheu-Gruttadauria, J. & MacRae, I. J. Structural basis for microRNA targeting. *Science* **346**, 608-613, doi:10.1126/science.1258040 (2014).

3 Humphrey, W., Dalke, A. & Schulten, K. VMD: visual molecular dynamics. *J Mol Graph* **14**, 33-38, 27-38, doi:10.1016/0263-7855(96)00018-5 (1996).

4 Huang, J. & MacKerell, A. D. CHARMM36 all-atom additive protein force field: Validation based on comparison to NMR data. *Journal of Computational Chemistry* **34**, 2135-2145, doi:10.1002/jcc.23354 (2013).

5 Darden, T., York, D. & Pedersen, L. Particle Mesh Ewald - an N.Log(N) Method for Ewald Sums in Large Systems. *Journal of Chemical Physics* **98**, 10089-10092, doi:Doi 10.1063/1.464397 (1993).

6 Phillips, J. C. *et al.* Scalable molecular dynamics with NAMD. *J Comput Chem* **26**, 1781-1802, doi:10.1002/jcc.20289 (2005).

7 Jorgensen, W. L., Chandrasekhar, J., Madura, J. D., Impey, R. W. & Klein, M. L. Comparison of Simple Potential Functions for Simulating Liquid Water. *Journal of Chemical Physics* **79**, 926-935, doi:Doi 10.1063/1.445869 (1983).

8 Bakan, A. *et al.* Evol and ProDy for bridging protein sequence evolution and structural dynamics. *Bioinformatics* **30**, 2681-2683, doi:10.1093/bioinformatics/btu336 (2014).

9 Valsson, O., Tiwary, P. & Parrinello, M. Enhancing Important Fluctuations: Rare Events and Metadynamics from a Conceptual Viewpoint. *Annu Rev Phys Chem* **67**, 159-184, doi:10.1146/annurev-physchem-040215-112229 (2016).

10 Fiorin, G., Klein, M. L. & Henin, J. Using collective variables to drive molecular dynamics simulations. *Mol Phys* **111**, 3345-3362, doi:10.1080/00268976.2013.813594 (2013).

11 Sanner, M. F., Olson, A. J. & Spehner, J. C. Reduced surface: An efficient way to compute molecular surfaces. *Biopolymers* **38**, 305-320, doi:Doi 10.1002/(Sici)1097-0282(199603)38:3<305::Aid-Bip4>3.3.Co;2-8 (1996).
