# Supplementary material for: Germline AGO2 mutations impair RNA interference and human neurological development
Source: Nat Commun. 2020 Nov 16;11:5797. doi: 10.1038/s41467-020-19572-5 (PMC7670403; doi:10.1038/s41467-020-19572-5)
Supplement: Supplementary file 3 — Reporting Summary [file 41467_2020_19572_MOESM3_ESM.pdf]

nature research

Corresponding author(s): *Double-blind peer review submissions: write DBPR and your manuscript number here instead of author names.*

Last updated by author(s): *YYYY-MM-DD*

Reporting Summary

Nature Research wishes to improve the reproducibility of the work that we publish. This form provides structure for consistency and transparency in reporting. For further information on Nature Research policies, see our Editorial Policies and the Editorial Policy Checklist.

Statistics

For all statistical analyses, confirm that the following items are present in the figure legend, table legend, main text, or Methods section.

n/a	Confirmed	
☐	☒	The exact sample size (*n*) for each experimental group/condition, given as a discrete number and unit of measurement
☐	☒	A statement on whether measurements were taken from distinct samples or whether the same sample was measured repeatedly
☐	☒	The statistical test(s) used AND whether they are one- or two-sided<br>*Only common tests should be described solely by name; describe more complex techniques in the Methods section.*
☒	☐	A description of all covariates tested
☐	☒	A description of any assumptions or corrections, such as tests of normality and adjustment for multiple comparisons
☐	☒	A full description of the statistical parameters including central tendency (e.g. means) or other basic estimates (e.g. regression coefficient) AND variation (e.g. standard deviation) or associated estimates of uncertainty (e.g. confidence intervals)
☐	☒	For null hypothesis testing, the test statistic (e.g. F, t, r) with confidence intervals, effect sizes, degrees of freedom and P value noted<br>*Give P values as exact values whenever suitable.*
☒	☐	For Bayesian analysis, information on the choice of priors and Markov chain Monte Carlo settings
☒	☐	For hierarchical and complex designs, identification of the appropriate level for tests and full reporting of outcomes
☒	☐	Estimates of effect sizes (e.g. Cohen's *d*, Pearson's *r*), indicating how they were calculated

Our web collection on statistics for biologists contains articles on many of the points above.

Software and code

Policy information about availability of computer code

Data collection	All MD simulations were conducted in NAMD 2.12 using CHARMM36 force field in TIP3P water box. Production MD simulations were performed in NVT ensemble using Langevin thermostat at standard conditions with 2 fs integration step. Gene ontology enrichment analysis for molecular function and biological process were obtained using the DAVID tool v6.8. Microscopic images were quantitatively analysed using ImageJ.
Data analysis	Data were analysed using Prism software (GraphPad, San Diego, CA).

For manuscripts utilizing custom algorithms or software that are central to the research but not yet described in published literature, software must be made available to editors and reviewers. We strongly encourage code deposition in a community repository (e.g. GitHub). See the Nature Research guidelines for submitting code & software for further information.

Data

Policy information about availability of data

All manuscripts must include a data availability statement. This statement should provide the following information, where applicable:

- Accession codes, unique identifiers, or web links for publicly available datasets
- A list of figures that have associated raw data
- A description of any restrictions on data availability

Data availability: All data needed to evaluate the conclusions in the paper are present in the paper and/or the supplementary materials. The RNA-seq data were submitted to GEO repository under the accession number GSE141099 [https://www.ncbi.nlm.nih.gov/geo/query/acc.cgi?acc=GSE141099]. The mass spectroscopy data for quantification of AGO2 phoshorylation have been submitted to the peptideatlas repository under accession PASS01561 [http://www.peptideatlas.org/PASS/PASS01561]. The raw whole-exome sequencing and microarray-based comparative genomic hybridization data that support the findings in affected individual cannot be made publicly available for reasons of patient confidentiality. Qualified researchers may apply for access to these data, pending institutional review board

approval. Cells are available upon signing a material transfer agreement. Source data are available with this paper. The source data underlying Figs 2, 3, 5c-e and Supplementary Figs 4-8, 10 and 17 are provided as a Source Data file.

Field-specific reporting

Please select the one below that is the best fit for your research. If you are not sure, read the appropriate sections before making your selection.

☒ Life sciences ☐ Behavioural & social sciences ☐ Ecological, evolutionary & environmental sciences

For a reference copy of the document with all sections, see nature.com/documents/nr-reporting-summary-flat.pdf

Life sciences study design

All studies must disclose on these points even when the disclosure is negative.

Sample size	Sample sizes were chosen to reliably measure experimental parameters while keeping with standards in the relevant fields, and remaining in compliance with ethical guidelines to minimize the number of animals used.
Data exclusions	no data were excluded from analysis.
Replication	The number and type of repeats for each experiment is described in the corresponding Figure legend.
Randomization	Animal subjects were not randomly allocated to experimental groups, as all comparisons were between identical genotypes.
Blinding	Experiments did not involve blinding.

Reporting for specific materials, systems and methods

We require information from authors about some types of materials, experimental systems and methods used in many studies. Here, indicate whether each material, system or method listed is relevant to your study. If you are not sure if a list item applies to your research, read the appropriate section before selecting a response.

Materials & experimental systems

n/a	Involved in the study	
☐	☒	Antibodies
☐	☒	Eukaryotic cell lines
☒	☐	Palaeontology and archaeology
☐	☒	Animals and other organisms
☒	☐	Human research participants
☒	☐	Clinical data
☒	☐	Dual use research of concern

Methods

n/a	Involved in the study	
☒	☐	ChIP-seq
☒	☐	Flow cytometry
☒	☐	MRI-based neuroimaging

Antibodies

Antibodies used	The following primary antibodies were used: Chicken anti-MAP2 (antibodies Online; ICC: 1:1000); guinea pig anti-Shank3 (Synaptic Systems # 162 304; ICC: 1:500); rabbit monoclonal anti Dcp1a, Abcam #183709; ICC 1:1000); rabbit anti-Shank and anti- mRFP antisera have been generated by custom immunization by Biogenes GmbH, Berlin, Germany. Anti-Dicer antibody (Bethyl, 1:1000), anti-HA antibody (Covance Research Products, clone 16B12, 1:1000) and anti-TNRC6ABC, clone 7A9 (Merck Millipore) were used for protein detection in Western Blotting. Secondary antibodies: Alexa fluor 633 goat anti-rb, Alexa 405 goat anti-chk IgG (abcam), Abberior star red goat anti guinea pig were used at 1:1000 dilution.
Validation	All antibodies are validated for detecting rat or human proteins by the manufacturer. Additionally, antibodies were in house confirmed for each specific application using cells of known origin that are known to express the antigen.

Eukaryotic cell lines

Policy information about cell lines

Cell line source(s)	Cell lines were obtained from the ATCC. Primary fibroblasts were generated in house.
Authentication	None of the cells have been authenticated.
Mycoplasma contamination	All cell lines were negative for mycoplasma.

Commonly misidentified lines (See ICLAC register)	None of the cells belongs to the ICLAC registry.

Animals and other organisms

Policy information about studies involving animals; ARRIVE guidelines recommended for reporting animal research

Laboratory animals	Rattus norwegicus; female pregnant animals were sacrificed, and all embryos used for preparation of cultures, regardless of sex.
Wild animals	no wild animals were used
Field-collected samples	This study did not involve field collection
Ethics oversight	All animal experiments were approved by, and conducted in accordance with, the guidelines of the Animal Welfare Committee of the University Medical Center (Hamburg, Germany) under permission number Org766.

Note that full information on the approval of the study protocol must also be provided in the manuscript.
