# Supplementary material for: Germline AGO2 mutations impair RNA interference and human neurological development
Source: Nat Commun. 2020 Nov 16;11:5797. doi: 10.1038/s41467-020-19572-5 (PMC7670403; doi:10.1038/s41467-020-19572-5)
Supplement: Supplementary file 4 — Description of Additional Supplementary Files [file 41467_2020_19572_MOESM4_ESM.pdf]

Description of Additional Supplementary Files

File Name: Supplementary Data 1
Description: Clinical characteristics of individuals with *AGO2* mutations.

File Name: Supplementary Data 2
Description: List of 485 commonly differentially-expressed genes in cases 2 and 3 and its fold-changes.

File Name: Supplementary Data 3
Description: Gene ontology analysis of the differentially-expressed genes in cases 2 and 3. Data were obtained using DAVID tool v.6.8. p-values were calculated using the modified Fisher's exact test. To globally correct enrichment p values, multiple testing correction techniques Bonferroni, Benjamini and FDR were used.

File Name: Supplementary Data 4
Description: List of 164 commonly differentially-expressed genes and its fold-changes in cases 2, 3 and 14.

File Name: Supplementary Data 5
Description: Data were obtained using DAVID tool v.6.8. p-values were calculated using the modified Fisher's exact test. To globally correct enrichment p values, multiple testing correction techniques Bonferroni, Benjamini and FDR were used.

File Name: Supplementary Movie 1
Description: Partial unwinding of the guide-target duplex facilitated by helix7 from the 1D MetD trajectory of WT g2-7 holo-RISC state. Color code: Helix 7 – yellow, guide RNA – green and target RNA – red. Both plots show the time evolution of the mean g2-g6 duplex width () (bottom) and a current position of a trajectory frame in the a7-MID; g(6-7) kink space (top).
